# Advancing biomolecular condensates research in Brazil: a collective effort

**DOI:** 10.1007/s12551-026-01454-7

**Published:** 2026-09-14

**Authors:** Aline Ribeiro Passos, Antonio Jose Costa-Filho, Carolina Gimenes Oliveira, Emanuel Kava, Guilherme Augusto Piedade de Oliveira, Ivan Rosa e Silva, Jerson Lima Silva, Juliana Ferreira de Oliveira, Kátia Maria dos Santos Cabral, Luis Felipe Santos Mendes, Marcius Da Silva Almeida, Mariana Juliani Do Amaral, Mayra Amorim Marques, Yraima Cordeiro

**Affiliations:** 1 Brazilian Synchrotron Light Laboratory (LNLS), Brazilian Center for Research in Energy and Materials (CNPEM), Campinas, SP Brazil; 2 Molecular Biophysics Laboratory, Department of Physics, Faculty of Philosophy, Sciences, and Literature at Ribeirão Preto, Universidade de São Paulo, Ribeirão Preto, SP Brazil; 3 National Institute of Science and Technology in Innovative Research in Health Sciences: From Nanotechnology to Artificial Intelligence, Ribeirão Preto, SP Brazil; 4 Group of Biophysics and Structural Biology “Sergio Mascarenhas”, São Carlos Institute of Physics, Universidade de São Paulo, São Carlos, SP Brazil; 5 Institute of Medical Biochemistry Leopoldo de Meis, Universidade Federal do Rio de Janeiro, Rio de Janeiro, RJ Brazil; 6 Laboratory of Structural Bioengineering Jerson Silva, Institute of Medical Biochemistry Leopoldo de Meis, Universidade Federal do Rio de Janeiro, Rio de Janeiro, RJ Brazil; 7 Center for Research in Energy and Materials (CNPEM), Brazilian Biosciences National Laboratory (LNBio), Campinas, SP Brazil; 8 D’Or Institute for Research and Education (IDOR), Rio de Janeiro, RJ Brazil; 9 Protein Advanced Biochemistry Laboratory, National Center for Structural Biology and Bioimaging (CENABIO), Universidade Federal do Rio de Janeiro, Rio de Janeiro, RJ Brazil; 10 National Institute of Science and Technology for Structural Biology, Universidade Federal do Rio de Janeiro, Rio de Janeiro, RJ Brazil; 11 Faculty of Pharmacy, Universidade Federal do Rio de Janeiro, Rio de Janeiro, RJ Brazil

**Keywords:** Biomolecular condensation, Phase separation, Membraneless organelles, Brazilian Biophysical Society (SBBf), Pathogenesis, Intrinsically disordered proteins

## Abstract

The ability of proteins to form scaffolds of biomolecular condensates through phase separation has emerged as an important physicochemical mechanism in cell biology, involved in a myriad of biological functions. Condensates contain highly concentrated biomolecules that function as hubs for nucleic acid storage and processing, while also enabling finely tuned spatiotemporal cellular organisation. Mounting evidence suggests that the transition of condensates from dynamic liquid-like states to gel- and solid-like structures underlies the pathogenesis of many degenerative diseases. Therefore, controlling phase behaviour has emerged as a potentially targetable mechanism for intervening in currently incurable proteinopathies. Over the last four decades, several Brazilian groups that now contribute to research on biomolecular condensates have built strong foundations in the biophysics of intrinsically disordered proteins, protein misfolding, nucleic acid interactions, and structural biology. A new phase for our community began to emerge around 2017, following insightful exchanges of ideas in which the Brazilian Biophysical Society (SBBf) played a key role. Here, we present a review of Brazilian studies focusing on biomolecular condensation, discussing their main findings, experimental approaches, open questions, and interdisciplinary opportunities in the field. This review also celebrates the 90th anniversary of SBBf, a society that has greatly fostered the scientific growth of Brazilian and Latin American scientists studying phase separation. We hope that the Brazilian studies highlighted here will inspire the next generation of scientists and galvanise cutting-edge research, helping to build and strengthen a broader biomolecular condensate research community in Brazil.

## Introduction

Biomolecular condensates provide a unifying framework for how cells can organise chemistry without membranes. They emerge from collective, multivalent interactions among biomolecules such as proteins, RNAs, and cofactors, giving rise to cellular assemblies ranging from ~ 20 nm to a few micrometres that can form and dissolve rapidly (Alberti et al. 2025; Roden and Gladfelter 2021). Condensate-forming proteins form a dense phase with concentrations 300-1,000 times higher than that of the diluted phase, leading to complex biophysical and biochemical properties (Farag et al. 2022; Galvanetto et al. 2023; Turoverov et al. 2019). This highly crowded local microenvironment of the condensate enables the selective concentration of specific components and the driving or suppression of reactions, thereby modulating both enzymatic and nonenzymatic reaction rates (e.g., spontaneous nonenzymatic reductive amination). Therefore, they contribute to signalling, gene regulation, stress responses, secretion, and many other biological activities (Alberti and Hyman 2021; He et al. 2020; Laflamme and Mekhail 2020; Lafontaine et al. 2021; Mangiarotti et al. 2023b; Nixon-Abell et al. 2025; Song et al. 2026; Zhang and Seemann 2024; Zhou et al. 2025). Indeed, condensates provide unique advantages, such as precise spatiotemporal control of cellular reactions. Over the last decade, the field expanded beyond describing “droplets” to address a broader problem: how molecular interactions generate specific material states and how those states influence or compromise biological function. This shift was naturally driven by mounting research revealing a variety of emergent physicochemical properties in condensates, placing mechanism and material state at the centre of the field and reframing condensates as an inherently integrative research area (Alberti et al. 2025).

Here, biomolecular condensates refer to mesoscale assemblies enriched in specific biomolecules, formed through collective interactions and displaying behaviours consistent with phase transitions or related condensation processes. The term includes liquid-like assemblies often associated with liquid–liquid phase separation (LLPS), as well as viscoelastic gels, glassy states, and solid-like structures (Fuxreiter and Vendruscolo 2021; Jawerth et al. 2020; Pappu et al. 2023). This inclusive definition is essential as many cellular assemblies do not form or maintain ideal liquid properties. Instead, they populate a continuum of physical states whose material properties can (and commonly do) change over time, varying across subcellular compartments, and depend on many different regulatory mechanisms (Cheng et al. 2025; Feric et al. 2016; King et al. 2024; Putnam et al. 2019). Consequently, the central question is not whether condensation occurs, but rather which material state is formed, how this process is (dys)regulated, and its associated pathophysiological consequences.

Three major features make biomolecular condensates intrinsically challenging to study across diverse disciplines and, therefore, community-level in scope. First, condensates are multiscale. At the molecular level, sequence composition, charge patterning, aromatic content, and interaction motifs determine valency and specificity (Wang et al. 2018). Post-translational modifications can rewire these interaction networks and shift condensation thresholds (Hofweber and Dormann 2019). At the mesoscale, multicomponent mixtures exhibit emergent behaviour, selective partitioning, compositional heterogeneity, and nontrivial phase diagrams that cannot be inferred from single affinities (Banani et al. 2017; Feric et al. 2016). At the cellular scale, condensates are coupled to transport, local synthesis and degradation, and active processes that maintain them out of equilibrium (Coupe and Fakhri 2026; Zwicker et al. 2014). Linking these scales requires integrated experimental design: mutational logic and biochemistry to define interaction rules, controlled reconstitution to test sufficiency, and quantitative cell measurements to assess physiological relevance (Alberti et al. 2025).

Second, condensates are, by composition, context-dependent. In vitro, phase behaviour is sensitive to salt, pH, polyvalent ions, temperature, macromolecular crowding, and the presence/nature of nucleic acids (Alberti et al. 2025; Silva et al. 2023). On the other hand, the cellular *milieu* is much more complex, as local concentrations and crowding are regulated, components are continuously modified, and surfaces and structures shape assembly pathways. Biological membranes are clear examples. Condensation at membranes can involve two-dimensional confinement, electrostatic recruitment to charged lipids, curvature or tension effects, and surface-assisted nucleation. All of these factors alter both thermodynamics and kinetics relative to bulk phase separation (Mangiarotti et al. 2023a, b; Su et al. 2025). RNAs can act as scaffolds, buffers, or switchers depending on length, structure, and binding landscape (Maharana et al. 2018). Metal ions and small molecules can promote or suppress condensation through charge neutralisation, coordination chemistry, or competition for key motifs (do Amaral et al. 2023; Sołtys et al. 2023). Taking the microenvironment into account as part of the mechanism, rather than as an annoyance variable, is therefore mandatory.

Third, the field still faces challenges in measurement and the establishment of standards. Many commonly used “condensate-like hallmarks”, such as rounded morphology, fusion, or fluorescence recovery after photobleaching (FRAP), are informative but not definitive (Alberti et al. 2019; Taylor et al. 2019). Exchange dynamics can be slow due to binding kinetics or viscoelasticity without implying a solid state. On the other hand, apparent liquidity can reflect only a subset of components or transient regimes (Mittag and Pappu 2022). In cell models, overexpression, artificial fluorescent constructs, probe perturbations, phototoxicity, and limited microscopy resolution can further confuse interpretation (Alberti et al. 2025). For this reason, our Brazilian community increasingly converges on multi-criterion evidence: concentration dependence, perturbations of valency or interaction motifs, sensitivity to environmental variables, orthogonal readouts of internal dynamics, and, when possible, quantitative mapping of phase boundaries. Establishing robust criteria is not a technical addendum. It shapes what is considered a *bona fide* condensate and how mechanistic claims can be compared across systems and laboratories.

With these criteria in place, the central question becomes mechanism-to-function (or dysfunction), rather than phenomenology. Condensation can accelerate reactions by locally concentrating enzymes and substrates, but it can also filter components, promote gate signalling, spatially pattern gene regulation, or create microenvironments with distinct solvent properties (Dustin and Muller 2016; O'Flynn and Mittag 2021). At the same time, the same multivalent networks that enable reversible assembly can drift toward kinetic traps and persistent states, particularly under stress, ageing, or disease-linked mutations (Gui et al. 2019; Michaels et al. 2023; Patel et al. 2015; Ray et al. 2020). The connection between liquid condensation and amyloid formation has been demonstrated across diverse eukaryotic systems. In metazoan disease-associated proteins, FUS condensates can progressively mature into aggregated states, a transition accelerated by ALS-linked mutations (Patel et al. 2015; Shen et al. 2023), while α-synuclein phase separation can promote amyloid nucleation and fibril formation (Ray et al. 2020). In yeast, the prion domain of the translation termination factor Sup35 drives reversible phase separation under stress, providing a protective state that can precede or compete with irreversible amyloid assembly (Franzmann et al. 2018). Together, these studies show that condensation and amyloid formation are mechanistically connected but not synonymous. The challenges, henceforth, are to define the variables that govern transitions along the liquid-gel-solid continuum and to determine when those transitions are adaptive rather than deleterious.

This review highlights contributions from Brazilian groups to research on biomolecular condensates within the broader international landscape. These contributions span neurobiology, infectious disease, protein misfolding, membrane-associated regulation, and the secretory pathway, supported by expertise in molecular biophysics, spectroscopy, structural biology, quantitative imaging, and bioinformatics. Such diversity is well-suited to the conception of biomolecular condensation as a cross-disciplinary subject: different groups addressing complementary pieces of the same multiscale puzzle, from molecular interaction rules to cellular function and material-state transitions. Rather than compiling a catalogue, our goal is to reveal how these contributions converge on shared conceptual challenges and, collectively, strengthen the field.

## Brazilian contributions to biomolecular condensates research

Although the studies discussed below emerged from distinct biological questions and experimental traditions, they converge on a set of shared conceptual themes. These include the regulation of condensation by nucleic acids, metal ions, post-translational modifications, and environmental variables; the transition of condensates across liquid-, gel-, and solid-like states; the coupling between condensation, protein misfolding, and disease; the influence of membranes and cellular interfaces; and the development of quantitative approaches to characterise condensate composition, dynamics, and material properties. We therefore discuss the Brazilian contributions not only as individual research trajectories, but also as complementary advances toward a mechanistic understanding of biomolecular condensation.

### Biomolecular condensates formed by the Brain Expressed X-linked Protein 3 (Katia Cabral, Marcius Almeida, and Mariana J. do Amaral)

Studies reported in the Master's thesis of Mariana do Amaral, supervised by Marcius Almeida and Katia Cabral at the Federal University of Rio de Janeiro, provided the earliest reported Brazilian experimental contributions to biomolecular condensation. The work focused on human Brain Expressed X-linked 3 (hBEX3), a neuronal protein involved in NGF-related signalling whose structure and assembly behaviour were still poorly understood. The BEX family is composed of five members (BEX1-BEX5), in which BEX3 stands out for its role as a downstream effector of the nerve growth factor (NGF) pathways. Interestingly, BEX proteins are specific to placental mammals, pointing to a highly specialised function for these proteins, which have been regarded as “signalling hubs” because they participate in many intracellular pathways (Fernandez et al. 2015). These proteins are predicted to exhibit a high degree of intrinsic disorder, and some members, such as BEX1, exhibit nucleic acid-related functions, including transcriptional regulation (Accornero et al. 2017; Calvo et al. 2015; Han et al. 2005). It was unknown whether the nucleic acid‑binding property observed in other BEX family members also applied to human BEX3 (hBEX3), a protein that undergoes nucleocytoplasmic shuttling and contains both intrinsically disordered regions and a coiled‑coil domain.

Early biochemical characterisation of hBEX3 revealed an unexpected coupling between nucleic acid binding, conformational organisation and higher-order assembly. An unusual purification strategy involving heating of the bacterial lysate yielded two molecular species by size-exclusion chromatography: a compact, nucleic acid-bound form with a reduced hydrodynamic radius and a nucleic acid-free higher-order oligomer. The bound nucleic acids were identified as transfer RNA (tRNA) fragments, which markedly influenced hBEX3 folding and promoted a disorder-to-order transition. In addition, hBEX3 solutions gradually separated into two phases and formed a temperature-reversible hydrogel, suggesting an intrinsic propensity for supramolecular organisation.

These observations were later reinterpreted within the emerging framework of biomolecular phase separation, following discussions with Vladimir Uversky in 2017, and motivated experiments to test the hallmarks of biomolecular condensation experimentally. This included visualising liquid-like condensates using differential interference contrast microscopy and testing the temperature dependence and reversibility of the phase separation, both by visual observation of clouding in the solution and by solution nuclear magnetic resonance (NMR). Consistently, hBEX3 exhibited intrinsic disorder, a coiled-coil domain, and a polyampholyte nature as predicted by bioinformatics; these are important biochemical characteristics that drive biomolecular condensation. In vitro reconstitution using recombinant hBEX3 showed homotypic phase separation that was dissolved upon 10% 1,6-hexanediol addition, while tRNA fragments modulated hBEX3 phase separation and were associated with reduced amyloid formation (do Amaral et al. 2020). These small tRNA fragments were comprised, at least in their majority, of the anticodon loop as identified by LC-MS (do Amaral et al. 2020) and were further identified by RNA next-generation sequencing via collaboration with Accornero’s laboratory (unpublished data). Despite the extensive biophysical and phase separation characterisation of BEX3 produced in vitro, cellular evidence for endogenous BEX3 condensates and their regulation by RNAs is largely unknown. However, the participation of another BEX member (BEX1) in condensate formation has been investigated in cells. BEX1 has emerged as a condensate-forming protein, concentrating cytoskeletal components that contribute to tubulin polymerisation (Hibino et al. 2022). Cytoplasmic granules composed of BEX1 show liquid-like FRAP recovery in a human epithelial cell line and promote the formation of primary cilia (Hibino et al. 2022). Also, BEX1 functions as an mRNA-dependent effector that induces the expression of pathology-promoting genes during cardiomyopathy and viral responses (Accornero et al. 2017; Martens et al. 2022). Because BEX family members show long IDRs with low complexity and coiled-coils, it is likely that they share the property of biomolecular condensation (Fernandez et al. 2015).

**Fig. 1:**
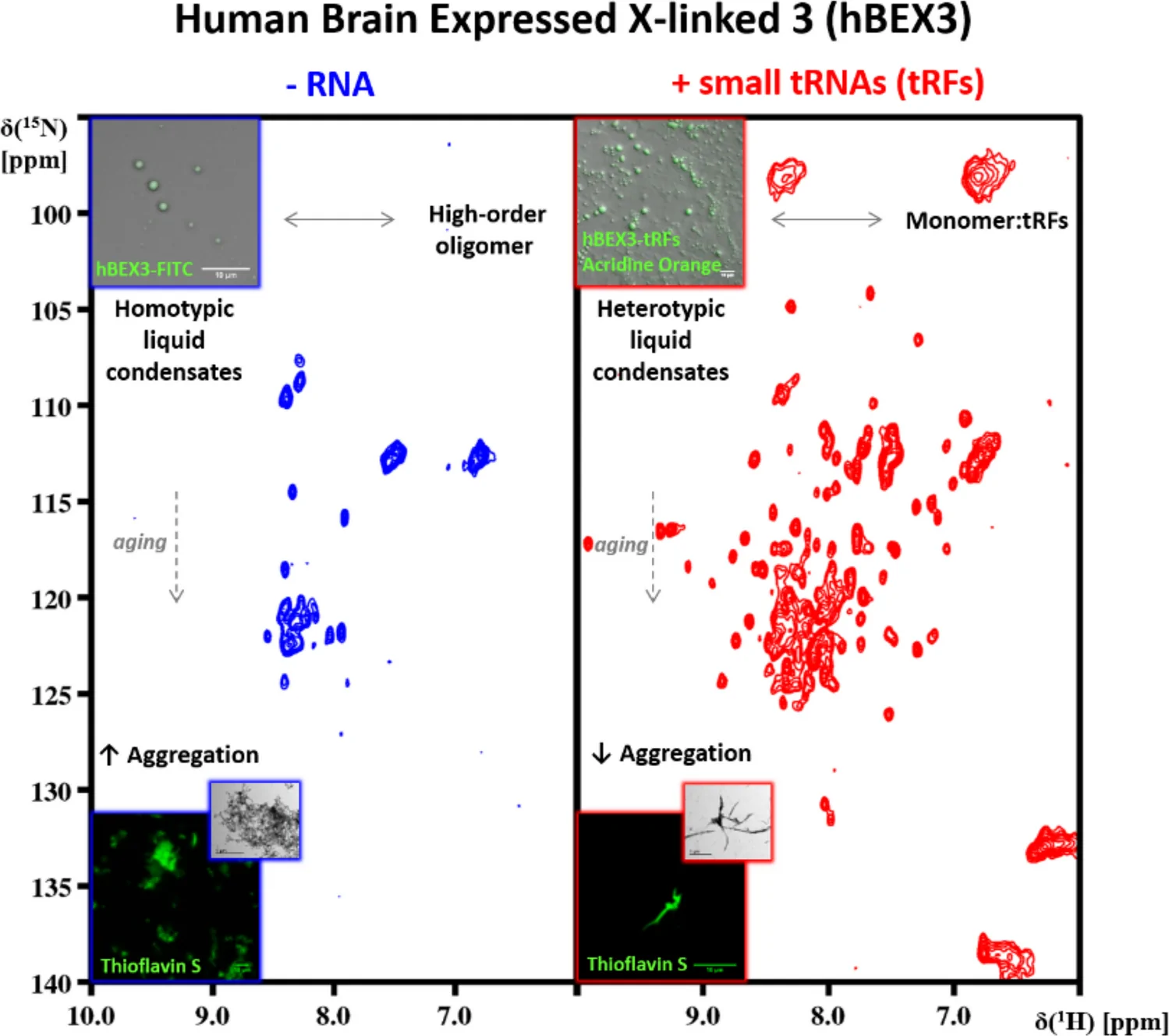
Phase Separation and Disorder-to-Order Transition of Human Brain Expressed X-Linked 3 (hBEX3) in the Presence of Small Fragments of tRNA. Human BEX3 is a disordered protein that assembles into liquid condensates in vitro (left). However, it co-purifies with small tRNA fragments (tRFs), which confer a well-defined 3D fold and a high number of small condensates (right). Over time, hBEX3 samples (kept in physiological buffer at 4 °C) transit from condensates to aggregates with morphology tuned by the presence of tRFs (fibrillary) or absence (amorphous). Aggregation is increased in the absence of tRFs, and in cells, tRFs might function as molecular chaperones, preventing the formation of misfolded higher-order hBEX3 species (do Amaral et al. 2020)

Despite the lack of cellular evidence for BEX3 condensation, the study by *do Amaral  *et al. 2021a, b was among the pioneering contributions from a Brazilian group to the emerging field of biomolecular condensation. The findings were originally reported in the author's Master's thesis, developed between 2016 and 2018 at the IBqM/UFRJ, and later published (do Amaral et al. 2020). The study established BEX3 as a model for investigating how intrinsic disorder, oligomerisation, and native tRNA fragments regulate condensate formation (Fig. 1).

Notably, prior Notably, prior to studies on human BEX3, the group had been investigating the structural and biophysical properties of mouse BEX3 since 2008. Early biophysical characterisation revealed that mBEX3 exhibits hybrid structural features, combining intrinsically disordered regions with organised coiled-coil elements. SAXS and AFM analyses showed that mBEX3 forms soluble high-order oligomers with a compact globular core surrounded by flexible peripheral regions. Complementary structural and denaturation studies further demonstrated the presence of proteolysis-resistant organised segments and non-cooperative unfolding behaviour, consistent with structurally heterogeneous intrinsically disordered proteins (Cabral et al. 2015).

Interestingly, recombinant mBEX3 was also observed to progressively transition into highly viscous, gel-like assemblies upon prolonged storage, suggesting an intrinsic propensity for self-association and supramolecular organisation (Cabral et al. 2015). Although these phenomena were originally interpreted within the framework of protein oligomerisation and partial intrinsic disorder, they are now remarkably consistent with phase-separating proteins and biomolecular condensates (Banani et al. 2017; Uversky 2017).

The BEX3 protein studies introduced important modulators that are shared by the Brazilian condensate studies: the ability of nucleic acids to trigger both protein conformational changes and phase separation and the possibility that dynamic assemblies mature into more solidified states. Interestingly, endogenous small tRNA fragments from bacteria that co-purified with BEX3 were able to modulate its phase transitions, highlighting possible physiological relevance, particularly given the involvement of other BEX family members in translational and transcriptional control (54). Building on these observations, research on PrP extends the role of nucleic acids in condensate regulation to a DNA-controlled reentrant phase transition, while further linking phase behaviour to protein misfolding. In other reconstituted systems, Cu(II) prevents PrP misfolding while promoting liquid-like phase separation, both in vitro and at cell-cell interfaces of live HEK cells, highlighting how environmental cues can differentially regulate condensation and pathological protein aggregation.

### Prion protein phase separation modulated by physiological ligands: insights from studies at the Federal University of Rio de Janeiro (Mariana Juliani do Amaral and Yraima Cordeiro)

#### PrP and nucleic acids

Aberrant phase transitions toward gel- or solid-like states have increasingly been associated with neurodegenerative diseases (Chakraborty and Zweckstetter 2023; Mukherjee et al. 2024). Among the proteins implicated in such transitions is the prion protein (PrP), which is involved in transmissible spongiform encephalopathies (TSEs), a group of progressive and fatal neurodegenerative disorders. Although relatively rare, these diseases impose a substantial burden on patients, with an average survival of approximately 18 months after the onset of clinical symptoms (Ascari et al. 2020; Prusiner 1998).

The hallmark of prion diseases is the conformational conversion of the cellular prion protein (PrP^C^), a soluble monomer enriched in α-helical structure, into a misfolded form known as scrapie PrP (PrP^Sc^), which is enriched in β-sheets and highly prone to aggregation (Cohen and Prusiner 1998; do Amaral and Cordeiro 2021b; Prusiner 1998). Although the molecular mechanisms underlying this conversion remain incompletely understood, experimental evidence indicates that molecular cofactors that bind to PrP can influence its conformational transitions (Cordeiro et al. 2023; do Amaral and Cordeiro 2021b; Supattapone 2020).

Among the proposed cofactors, nucleic acids (NAs) have attracted particular attention, as described in studies by Yraima Cordeiro’s group and collaborators (reviewed in Cordeiro et al. 2023; Macedo and Cordeiro 2017)). Their work has shown that nucleic acids interact with full-length recombinant PrP (rPrP^23−231^) and modulate its aggregation behaviour in vitro, potentially acting as catalysts or templates for structural conversion (Cordeiro et al. 2001; Gomes et al. 2008; Kovachev et al. 2019; Macedo et al. 2012). Both DNA and RNA can trigger conformational changes in PrP that depend on the protein:NA stoichiometry and on the structure of the nucleic acid ligand, leading to assemblies ranging from soluble complexes to aggregated or solid-like states (reviewed in (Cordeiro et al. 2023; do Amaral and Cordeiro 2021b), Fig. 2).

**Fig. 2:**
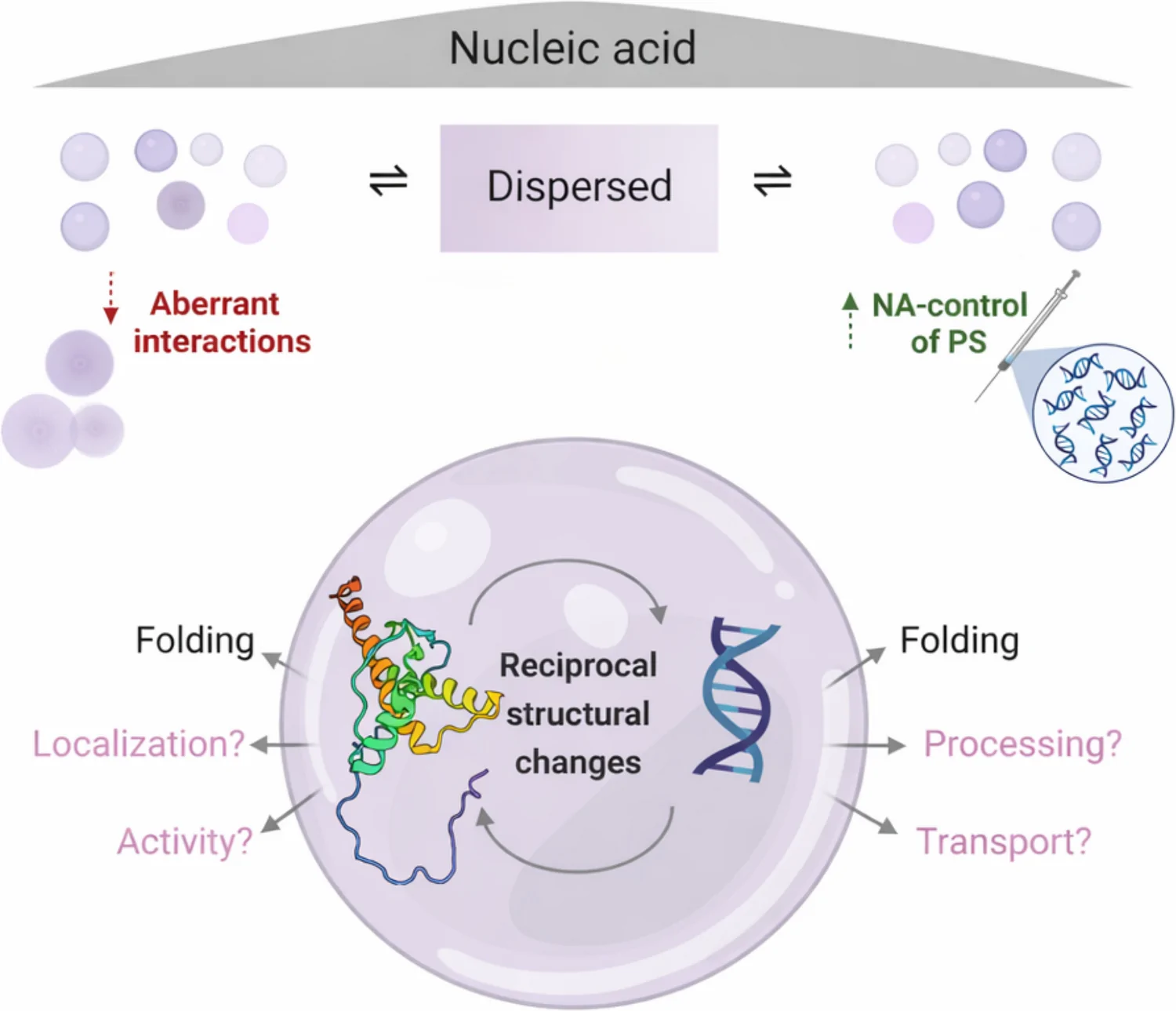
Phase separation of PrP modulated by nucleic acids. Based on Cordeiro’s laboratory in vitro data using recombinant (full-length and PrP^90−231^) (Matos et al. 2020; Tahira et al. 2025) and previous observations of PrP-containing stress granules (Goggin et al. 2008), we propose that PrP^C^ might assemble into or be recruited to biomolecular condensates in the presence of nucleic acids via a reentrant mechanism, as supported by in vitro data (Matos et al. 2020; Tahira et al. 2025). Varying nucleic acid:PrP stoichiometric ratios control condensate assembly or dissolution (top). Under cellular stress, these condensates may mature into solid-like states, and aberrant interactions with DNA or RNA might contribute to dysfunctional or pathological assemblies (top left). Whether pharmacological strategies aimed at modulating PrP condensation could be beneficial, and whether such approaches might exploit high-affinity DNA aptamers, remain largely unexplored (top right). Spectroscopic studies have demonstrated that nucleic acid binding induces conformational changes in soluble recombinant PrP, whereas PrP itself has been reported to promote nucleic acid unfolding, suggesting an interplay of reciprocal structural changes between both partners (do Amaral and Cordeiro 2021a, b) (bottom). Whether nucleic acids regulate PrP localisation and/or biological activity, and how PrP influences nucleic acid processing and intracellular transport are currently unknown. This hypothetical schematic model is based on in vitro data and highlights key questions that warrant further investigation. Created with BioRender.com

Over the past decade, the concept of phase separation has provided a useful framework for interpreting these observations. Several intrinsically disordered proteins associated with proteinopathies have now been characterised as undergoing phase separation, forming dynamic biomolecular condensates that can age into solid-like material. In this context, several groups have also investigated both homo- and heterotypic phase separation of different constructs of recombinant PrP (do Amaral et al. 2023; Kamps et al. 2021; Kostylev et al. 2018; Matos et al. 2020; Rai et al. 2023; Passos et al. 2021), demonstrating that diverse ligands, including nucleic acids, metal ions, and proteins or peptides, can modulate the formation and properties of PrP condensates.

Subsequent studies extended these observations by showing that both cellular RNAs and defined nucleic acid ligands regulated full-length PrP (rPrP^23−231^) phase behaviour (Gomes et al. 2008; Tahira et al. 2025). Total RNA extracted from neuroblastoma cells induced conformational rearrangements in recombinant PrP and promoted its transition from soluble species to phase-separated and aggregated assemblies. These findings support the idea that endogenous RNAs can act as physiologically relevant modulators of PrP condensation and aggregation (Gomes et al. 2008; Tahira et al. 2025). RNA molecules recovered from PrP-containing insoluble assemblies revealed a heterogeneous population of transcripts enriched for low-complexity sequence motifs, which are frequently present in ribosomal RNAs (Tahira et al. 2025). These results suggest that subsets of cellular RNAs sharing structural features can bind PrP and promote phase transitions in a stoichiometry-dependent manner, supporting the idea that endogenous nucleic acids may act as physiologically relevant modulators of PrP phase behaviour.

In parallel, DNA aptamers were later shown to modulate PrP phase separation in a conformation- and stoichiometry-dependent manner. Aptamers are structured nucleic acids selected for high-affinity binding to specific targets and can adopt diverse secondary and tertiary structures (Keefe et al. 2010). In the case of full-length PrP (rPrP^23−231^) and its extended C-terminal domain construct (rPrP^90−231^), DNA aptamers modulated phase separation in a conformation- and stoichiometry-dependent manner (Matos et al. 2020; Passos et al. 2021). Remarkably, different aptamer structures led to distinct outcomes: a hairpin aptamer promoted the formation of liquid droplets, whereas an unstructured single-stranded aptamer triggered transitions toward amyloid-like states of rPrP^90−231^ (Matos et al. 2020). These observations highlight the importance of ligand conformation in determining the material properties of PrP condensates, influencing whether assemblies remain reversible or progress toward aggregation.

#### Divalent metal ions trigger PrP condensate formation

Copper ions are known to bind to both recombinant (Thompsett et al. 2005) and cellular PrP (Brown et al. 1997) with high affinity (equilibrium dissociation constant, K_D_: 0.1 nM to 10 µM, depending on Cu^2+^ occupancy) (Walter et al. 2006), and in turn, Cu^2+^ modulates PrP^C^ expression (Varela-Nallar et al. 2006) and cellular localisation (Pauly and Harris 1998). PrP can bind Cu(II) via its N-terminal domain, which is intrinsically disordered and contains four repeats of the octapeptide sequence PHGGGWGQ (Brown et al. 1997). Many groups have suggested that PrP is a metalloprotein that plays a role in regulating brain copper homeostasis (Nguyen et al. 2019). Indeed, metal dyshomeostasis (*e.g.*, Cu^2+^, Zn^2+^, Fe^2+^, Mn^2+^) is implicated in the development of prion diseases (Wong et al. 2001), and a reduced cellular response to oxidative stress is observed in prion-infected cells and in the hippocampus of scrapie-infected mice (Johnson et al. 2013; Milhavet et al. 2000; Nguyen et al. 2019; Spiers et al. 2022).

Because Cu^2+^ is redox-active and can participate in Fenton reactions that produce reactive oxygen species (ROS), it has been suggested that PrP functions as a Cu^2+^ buffer, sequestering excess ions in the synaptic cleft and thereby exerting an antioxidant role in neurons (Juliani do Amaral et al. 2025). However, the molecular mechanism underlying PrP metal-ion sequestration remained unknown.

*Do Amaral *et al. 2021a, 2024b provided evidence that PrP^C^ condensates efficiently sequester copper ions, most likely at cell membrane interfaces (do Amaral et al. 2023). In HEK293 cells, Cu^2+^ promoted PrP^C^ enrichment at cell–cell junctions, suggesting that copper-driven condensation can cooperatively recruit PrP molecules to membrane-associated sites. Next, the effects of Cu^2+^ on recombinant PrP^23−231^ condensation and aggregation at physiologically relevant concentrations of PrP, ionic strength, pH, and molecular crowding were dissected, providing evidence for the role of PrP:Cu^2+^ condensation in a cellular context. Cu^2+^ prevented the misfolding and amyloid aggregation of recombinant full-length PrP. However, oxidation induced by Cu^2+^/H_2_O_2_ promoted the maturation of PrP condensates through a liquid-to-solid transition. Such oxidative stress is associated with ageing and prion disease progression. A mechanism was identified in which Cu^2+^-driven PrP condensation buffers excess redox-active copper ions, revealing how oxidation via aberrant phase separation triggers PrP aggregation (do Amaral et al. 2023).

Given that PrP also coordinates zinc ions in the N-terminal octarepeat domain, albeit with lower affinity than Cu^2+^ (K_D_ of 20–200 µM) (Millhauser 2023), subsequent work investigated whether Zn^+2^ also triggers PrP phase separation. The results show that Zn^2+^ promoted phase separation of rPrP^23−231^ with increasing physiological concentrations. Still, the maximum phase separation propensity required approximately six times as many zinc ions as copper ions. Moreover, FRAP showed that PrP:Cu^2+^ condensates were more densely packed than PrP:Zn^2+^ condensates, supporting the idea that intermolecular networks differ depending on the metal ion. Furthermore, while Cu^2+^-driven PrP condensates inhibited seeded conversion, Zn^2+^-driven condensates only delayed misfolding without fully preventing the formation of ThT-positive aggregates (Juliani do Amaral et al. 2025). These findings highlight distinct biophysical properties of PrP condensates depending on whether Cu^2+^ or Zn^2+^ is involved. Do Amaral’s laboratory is now studying whether other metal ions present in the synaptic cleft and intracellularly modulate PrP condensation, and their involvement with prion diseases and other neurodegenerative disorders.

While the data from PrP studies suggest how ligands and redox-active metal ions regulate the balance between protective condensation and pathological aggregation, work on Golgi proteins expanded the discussion to membrane-associated condensation and cellular organisation. Together, these systems illustrate how the biological consequences of condensation depend strongly on the physicochemical and microenvironment context in which the dense phase forms.

### Biomolecular condensates in Golgi biology and biotechnology: contributions from the University of São Paulo (Antonio Jose Costa-Filho and Luis Felipe Santos Mendes)

#### Phase separation in Golgi dynamics and unconventional protein secretion

The determinants of the architecture of the Golgi apparatus, organised as stacks of flattened cisternae, remain a major open question in cell biology. Classical models have emphasised vesicular trafficking, membrane tethering, and cytoskeletal organisation, but the physical principles that generate and maintain the Golgi´s shape remain unclear. An influential conceptual framework was proposed by James Rothman and colleagues, who suggested that the Golgi stack could behave as a phase-separated liquid crystal (Rothman 2019). This hypothesis placed Golgi matrix proteins at the centre of a broader physical problem: whether self-assembling protein networks could contribute directly to Golgi architecture. Studies from the University of São Paulo groups led by Antonio J. Costa-Filho and Luis Felipe S. Mendes have explored this possibility by investigating whether Golgi matrix proteins possess intrinsic features compatible with biomolecular condensation (Mendes et al. 2024b).

A large-scale bioinformatic analysis of more than 28,000 protein sequences showed that Golgi matrix proteins across the eukaryotic tree display a conserved propensity for phase separation (Mendes et al. 2024b). This tendency was associated with extensive intrinsically disordered regions and multivalent interaction surfaces, features that can support the formation of viscoelastic protein networks. These observations suggested that condensation is a broadly conserved property of Golgi-associated scaffolding proteins rather than an isolated feature of a few individual components. Experimental work on the GRASP family provided direct support for this model. Human GRASP55 was shown to form condensates with liquid-like properties, including fusion and internal molecular exchange. GRASP55 condensation was strongly pH-dependent, with maximal condensation around pH 6.0–6.3. Because GRASP55 is anchored to the cytoplasmic face of Golgi cisternae, this behaviour should not be interpreted as direct sensing of the acidic Golgi lumen. Instead, it reveals an intrinsic sensitivity of GRASP55 condensation to protonation state. Local proton activity at membrane interfaces can differ from that of the bulk cytosol, particularly at negatively charged lipid surfaces, raising the possibility that the membrane-associated environment contributes to this regulation (Mendes et al. 2018). Whether such interfacial effects are sufficient to modulate GRASP55 condensation at the Golgi remains to be established.

**Fig. 3:**
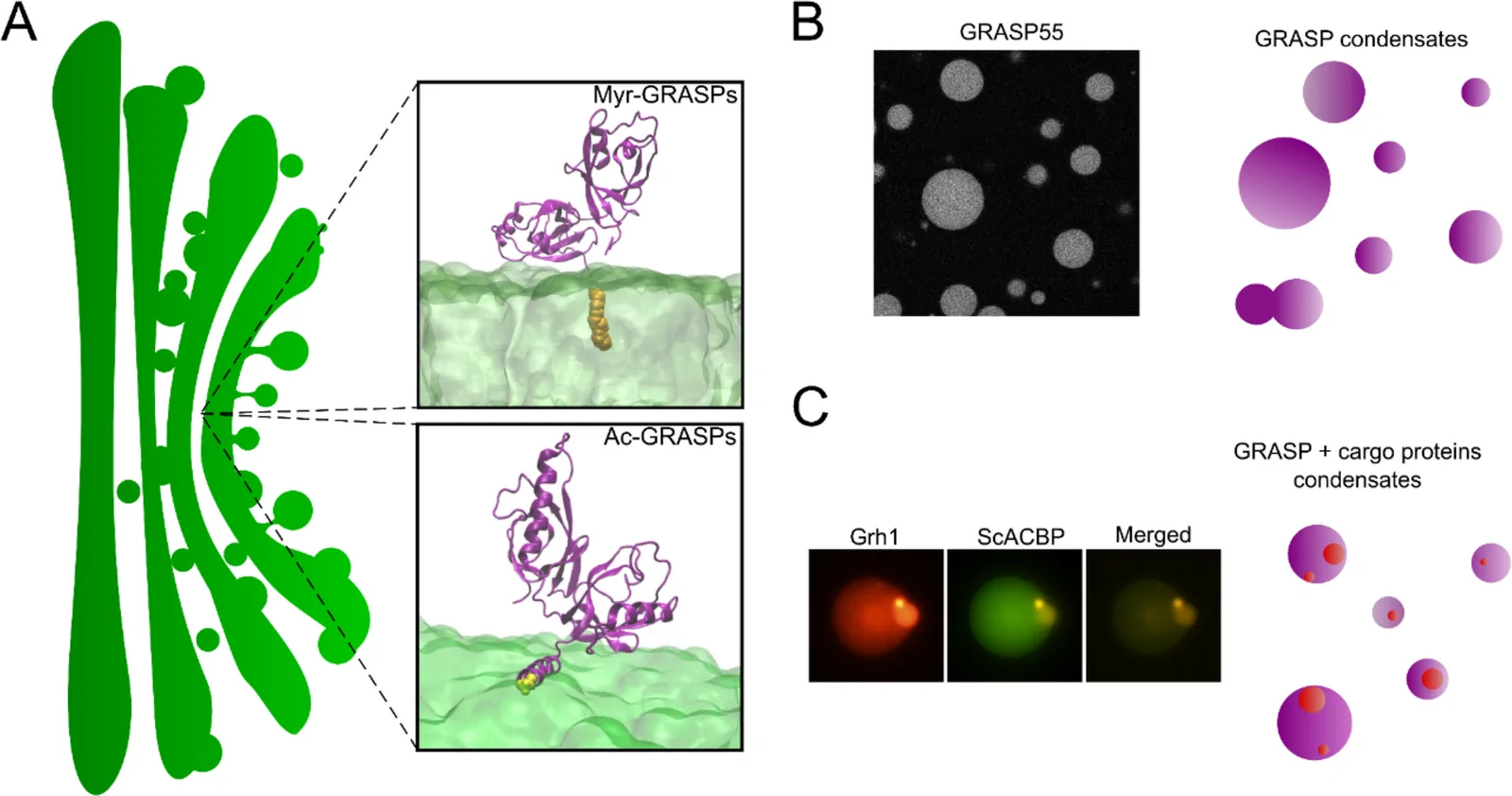
Membrane anchoring, phase separation, and cargo co-condensation by GRASP proteins. **A** Membrane interaction mechanisms of the GRASP family are mediated by either myristoylation or acetylation. In the upper panel, the myristoyl moiety is represented by the attached orange carbon chain; in the lower panel, the acetyl group is shown in yellow. **B** Phase separation of GRASPs is represented by a microscopy image of GRASP55 condensates (91). **C** Co-condensation of Grh1 and ACBP might suggest that GRASPs can interact with cargo proteins involved in unconventional secretion within biomolecular condensates, but this model remains to be further investigated (Mendes et al. 2024a)

Because the Golgi is a membrane-bound organelle, phase separation in this context cannot be fully understood in bulk solution alone. Condensation must be considered alongside membrane binding, lipid composition, bilayer mechanics, and interfacial organisation (Mangiarotti and Dimova 2026; Mangiarotti et al. 2023b). Recent work addressed this issue by reconstituting full-length myristoylated human GRASP55 and GRASP65 (Fig. 3), as well as their isolated GRASP domains, into model lipid membranes (Kava et al. 2026). This approach was important because previous in vitro studies often neglected the physiological lipid modification that anchors metazoan GRASPs to Golgi membranes. The results showed that myristoylation is not only a membrane-targeting signal but also a determinant of bilayer remodelling. Myristoyl-anchored GRASPs altered lipid packing, membrane dynamics, and interfacial hydration, with effects concentrated near the membrane interface. Full-length proteins produced stronger perturbations than isolated GRASP domains, indicating that the disordered serine-proline-rich region also contributes to membrane interaction. Together with recent evidence that condensate wetting can remodel lipid bilayers, these findings support a model in which GRASP condensation and membrane binding could be physically coupled processes. In this view, Golgi matrix proteins may contribute to organelle architecture not only by acting as molecular tethers but also by generating membrane-associated condensates that reshape local bilayer properties (Kava et al. 2026). Ongoing studies are investigating the molecular mechanisms and architectural consequences of this coupling.

#### The potential role of biomolecular condensates in unconventional secretion

Unconventional protein secretion provides a route for the extracellular release of proteins that bypass the classical ER–Golgi secretory pathway (Chen et al. 2025; Chiritoiu-Butnaru et al. 2022). This process becomes particularly relevant during cellular stress, starvation and proteostatic imbalance, when cells must redirect specific leaderless proteins toward noncanonical export routes (Filaquier et al. 2022). In parallel, biomolecular condensates have emerged as dynamic platforms that organise intracellular reactions, concentrate selected components and create local physicochemical environments distinct from the surrounding cytoplasm (Banani et al. 2017). These properties make phase separation a plausible mechanism for organising unconventional secretory compartments. In yeast, the GRASP homologue Grh1 is involved in the formation of the compartment for unconventional protein secretion (CUPS), a stress-induced structure associated with the secretion of leaderless cargoes (Bruns et al. 2011; Manjithaya et al. 2010).

Recent work proposed that phase separation might contribute to the biogenesis and functional organisation of CUPS. Under starvation-like conditions, cytosolic acidification promotes Grh1 condensation into liquid-like assemblies (Mendes et al. 2024a). These condensates are proposed to act as precursors or organising hubs for CUPS formation. A key observation is that the leaderless cargo protein ACBP shows limited affinity for soluble Grh1 but is efficiently enriched within condensed Grh1 assemblies (Fig. 3). This suggests that phase separation creates a distinct molecular environment that can favour cargo recruitment and selection. Therefore, Grh1 condensation may not simply reflect protein self-association under stress, but may actively participate in the spatial organisation of unconventional secretion.

The material state of Grh1 condensates is also context-dependent (Mendes et al. 2024a). Under physiological stress-related conditions, condensation appears to protect Grh1 from amyloid-like transitions by preventing access to aggregation-prone intermediate states. However, prolonged stress or environmental modulators such as heparin and specific ions can promote maturation into less dynamic, solid-like assemblies and eventually amyloid-like fibrils. This dual behaviour illustrates a broader principle in condensate biology: the same phase transition that supports adaptive organisation can, under altered conditions, shift toward persistent or pathological material states. These findings connect Golgi-related proteins, stress physiology, and condensate maturation in a single framework. They suggest that GRASP proteins can use phase separation to organise unconventional secretion, while also being susceptible to material-state transitions that influence their conformational fate (Mendes et al. 2024a).

#### N-terminal acetylation as a regulator of condensate material properties

Among post-translational modifications, N-terminal acetylation occupies a peculiar position: it is highly prevalent, often co-translational, and usually irreversible, yet its consequences for biomolecular condensation have received comparatively little attention. This is notable because N-terminal acetylation modifies the chemical identity of the protein N-terminus, neutralising its positive charge and introducing a more hydrophobic acetylated cap, with potential consequences for folding, protein–protein interactions, subcellular targeting, and protein stability (Mctiernan et al. 2025). Recent reviews have emphasised that post-translational modifications can regulate phase separation by altering charge distribution, multivalency, local structure, and intermolecular interaction networks, but most examples have focused on phosphorylation, methylation, ubiquitination or lysine acetylation (Liu et al. 2024; Mctiernan et al. 2025; Saito et al. 2019).

Recent work on the yeast GRASP protein Grh1 directly tested how N-terminal acetylation affects biomolecular condensation (G. Oliveira et al. 2026). By comparing acetylated and non-acetylated forms of the same full-length protein, the study showed that a single N-terminal chemical modification can substantially reshape phase behaviour. N-terminal acetylation increased the saturation concentration for condensation, reduced droplet number and size, dampened the response to acidic pH, weakened electrostatic contributions and altered the internal hydration of condensates. These effects indicate that N-terminal acetylation does not merely influence protein localisation or stability, but can also tune the mesoscale properties of condensates.

A particularly important finding was that acetylated and non-acetylated Grh1 are only partially miscible. When mixed, the two proteoforms formed core–shell condensates, consistent with acetylation-dependent changes in interfacial properties. This behaviour suggests that N-terminal acetylation can regulate not only whether condensation occurs, but also the internal organisation and compositional architecture of the dense phase. Mechanistically, these effects were linked to acetylation-dependent dimerisation and concentration-dependent structural rearrangements, including increased α-helical content at high protein concentration. This is relevant because Grh1 had already been recognised as a NatC-dependent Golgi-associated protein, and previous studies had connected N-terminal acetylation to Golgi localisation and membrane association (Cruz-Garcia et al. 2014; Levi et al. 2010). The new findings extend this view by showing that the same modification also regulates condensate material properties (Oliveira et al. 2026).

Beyond the specific case of Grh1, this study broadens the conceptual framework for how protein N-termini can influence phase behaviour. It suggests that co-translational modifications may preconfigure proteins for distinct condensate states before they encounter their cellular partners or membrane environments. The work is the first publication from the newly established group led by Luis F. S. Mendes at the São Carlos Institute of Physics, University of São Paulo, marking the beginning of an independent research program focused on protein disorder, phase separation, and Golgi biology.

#### Biomolecular condensates as synthetic microreactors

Research on biomolecular condensates has moved far beyond merely describing how cells organise their interiors without membranes. More recently, these systems have become attractive platforms for designing synthetic microcompartments and artificial organelles that control biochemical reactions in space and time (Banani et al. 2017; Bracha et al. 2019; Papp et al. 2025). Unlike membrane-bound compartments, condensates function as open, dynamic systems that selectively concentrate proteins, substrates, and cofactors, while creating local physicochemical environments distinct from the surrounding medium. This selective partitioning can strongly influence reaction rates, substrate accessibility, and even enzyme stability (Papp et al. 2025). A promising strategy for engineering these systems is to construct chimeric proteins that combine a structured catalytic domain with an intrinsically disordered, low-complexity domain (LCD), which serves as a molecular scaffold for phase separation (Faltova et al. 2018; Papp et al. 2025). These LCDs are typically enriched in repetitive polar, aromatic, and charged residues that promote weak, multivalent interactions, thereby allowing the formation of dense, protein-rich phases (Wang et al. 2018). In this context, condensates not only increase local concentration but also behave as a distinct reaction medium, modifying diffusion, local polarity, hydration, ionic strength, and pH. Therefore, they alter enzyme kinetics in ways not observed in dilute solution (Peeples and Rosen 2021).

This concept was recently explored in environmental biotechnology to develop a condensate-based microreactor for degrading toxic polycyclic aromatic hydrocarbons (PAHs) (Evangelista et al. 2024). The work, led by the group of Antonio Costa-Filho, focused on chlorocatechol 1,2-dioxygenase (Pp 1,2-CCD) from *Pseudomonas putida*, an enzyme that cleaves aromatic rings during the degradation of recalcitrant compounds. To induce phase separation, the enzyme was fused to LCD sequences derived from the DEAD-box protein Dhh1 and subjected to macromolecular crowding conditions. Adding the LCD sequences did not disrupt the structure of the enzyme’s catalytic core. Once the condensates formed, about 70% of the enzyme was concentrated in the dense phase, which remained permeable to external molecules, allowing the enzyme to preserve its catalytic activity. Over time, these condensates gradually changed from a more fluid, liquid-like state to more solid, irreversible assemblies. These solid-like assemblies were also active, making them particularly interesting for bioremediation applications, where enzymes are often exposed to harsh environmental conditions. By combining modular phase-separating domains with specific enzymatic functions, biomolecular condensates offer promising platforms for designing programmable microreactors that can organise enzymatic cascades, improve metabolic channelling, and enable more efficient approaches in green chemistry and environmental remediation.

The GRASP studies further demonstrate that condensates can function as adaptive organisational platforms. The p53 studies address the complementary pathological scenario, in which condensation becomes an intermediate toward aberrant aggregation and oncogenic gain of function.

### p53 condensation, oncogenic aggregation, and cancer: biophysical insights from UFRJ (Guilherme Augusto Piedade de Oliveira, Jerson Lima Silva, and Mayra Amorim Marques)

#### p53 at the interface between conformational plasticity, aggregation, and biomolecular condensation

The tumour suppressor p53 occupies an unusual position in cancer biology because its function cannot be understood from a single structural state. Encoded by *TP53*, p53 acts as a cellular stress integrator, translating DNA damage, oncogene activation, metabolic perturbation, hypoxia, ribosomal stress, and other insults into context-dependent transcriptional and non-transcriptional responses (Cañadillas et al. 2006; Joerger and Fersht 2008). In its canonical role, p53 preserves tissue integrity by inducing cell-cycle arrest, senescence, apoptosis, ferroptosis, metabolic rewiring, and DNA repair programs (Brady et al. 2011; Jiang et al. 2015; Valente et al. 2013). Yet this classical description captures only part of the problem. p53 is not a rigid transcription factor that simply binds DNA when activated. It is a multidomain, conformationally plastic protein whose biological output depends on how its domains, post-translational modifications, oligomeric states, and molecular partners are coupled in time and space. This is why *Marques, de Oliveira,* and *Silva* described p53 as a “molecular chameleon”: a protein able to redistribute its conformational ensemble according to cellular stimuli, but also one whose plasticity can be exploited pathologically in cancer (Marques et al. 2022).

**Fig. 4:**
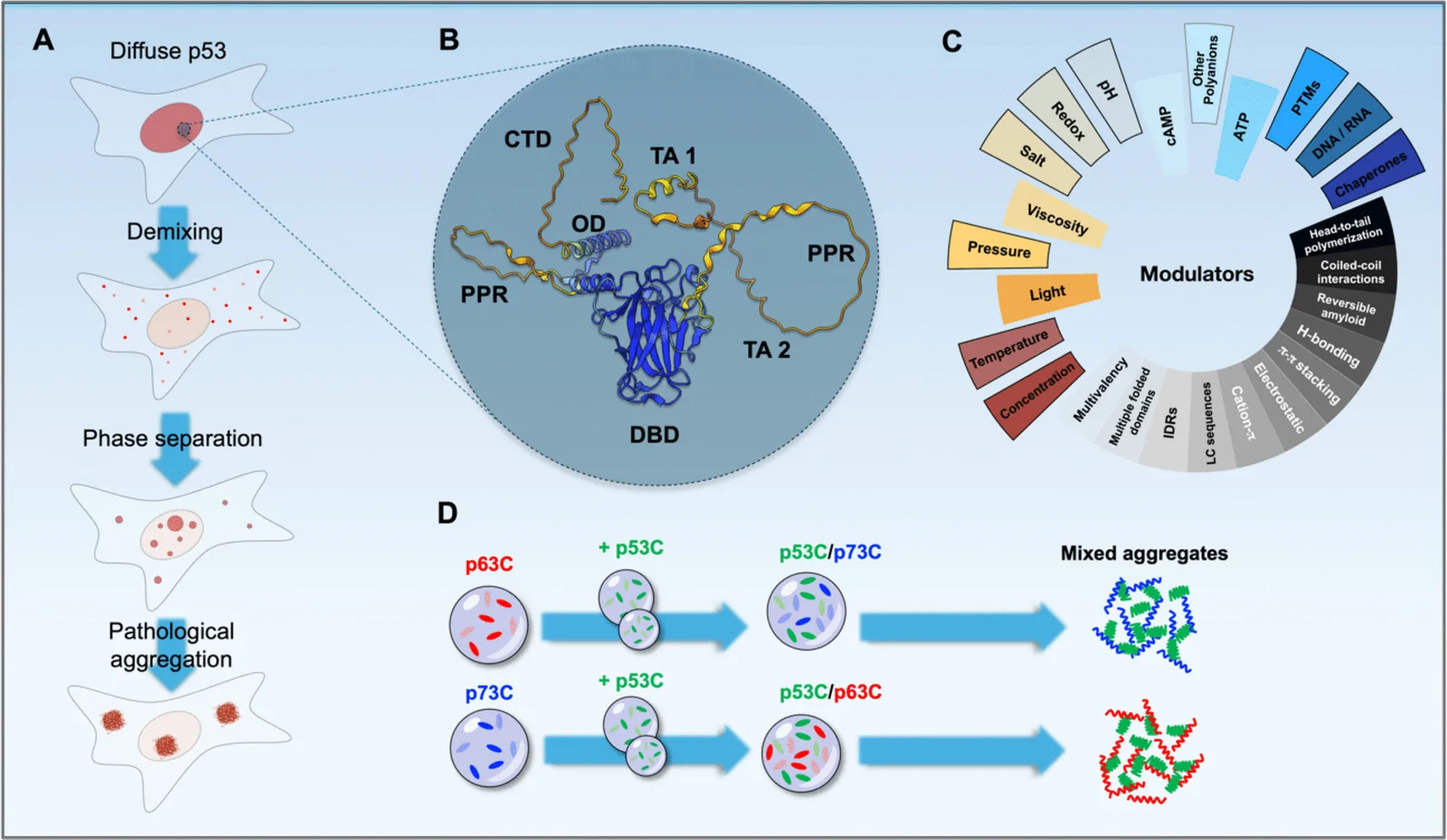
Multidomain architecture and regulatory landscape of p53 phase separation and aggregation. **A** Schematic representation of the progressive redistribution of p53 from a diffuse intracellular state to demixed condensates, phase-separated droplets, and ultimately pathological aggregates. **B** Domain organisation of p53, highlighting the intrinsically disordered transactivation regions TA1 and TA2, the proline-rich region/segments (PPR), the structured DNA-binding domain (DBD, or p53c), the oligomerisation domain (OD), and the C-terminal domain (CTD). The coexistence of folded and disordered regions provides multiple interaction surfaces that may contribute to multivalent contacts, condensate formation, and aggregation. **C** Overview of physicochemical, molecular, and sequence-dependent modulators that influence p53 phase behaviour. Among the diverse modulators shown, those positioned in the outermost ring represent factors that have already been experimentally demonstrated to drive p53 phase separation. **D** Proposed model for cross-interactions between p53, p63, and p73 core domains. Addition of p53C to p63C- or p73C-containing assemblies may promote the formation of mixed p53C/p63C or p53C/p73C condensates, which can further mature into mixed aggregates, suggesting a potential mechanism by which mutant or aggregation-prone p53 species may interfere with p53-family protein homeostasis. This model-based panel is schematic and not drawn to scale

The major cancer-associated mutations in *TP53* cluster in the DNA-binding domain (DBD, also called p53C). These mutations can impair p53 activity by at least three overlapping routes: loss of sequence-specific DNA binding, dominant-negative inhibition of the remaining wild-type allele, and gain-of-function activities that actively promote malignancy (Joerger and Fersht 2016). The biophysical basis of these phenotypes is not limited to altered DNA contacts. Many mutations destabilise the p53C, disrupt zinc coordination, expose aggregation-prone segments, or favour partially unfolded intermediates. Consequently, mutant p53 often accumulates in cancer cells not as a properly folded inactive protein, but as a population of non-native conformers that can assemble into higher-order structures. Earlier studies have shown that p53, particularly its DNA-binding domain, could form amyloid-like fibrils, oligomers, and amorphous aggregates, and that mutant p53 aggregates could be detected in tumour cells and tissues (Fig. 4a-d) (Ano Bom et al. 2012; Butler and Loh 2003; Pedrote et al. 2020; Xu et al. 2011). These aggregates were linked to the loss of tumour-suppressive function, dominant-negative behaviour, and oncogenic gain of function, including chemoresistance, altered proliferation, and aberrant interactions with other tumour suppressors (Silva et al. 2023).

This aggregation-centred view was essential, but it left a mechanistic gap: how does p53 transition from soluble monomers, dimers, or tetramers to amyloid-like material within the crowded and highly organised nuclear environment? The emerging field of biomolecular condensates offered a conceptual bridge. Biomolecular condensates are membraneless compartments formed by phase separation, in which biomolecules demix into dense and dilute phases (Alberti and Dormann 2019; Brangwynne et al. 2015). These assemblies can be liquid-like, dynamic, and reversible, but they can also age into gel-like, glassy, or solid-like states. In normal physiology, this behaviour allows cells to organise reactions without membranes, concentrate enzymes and substrates, buffer molecular interactions, and regulate processes such as transcription, RNA metabolism, DNA repair, and stress responses. In disease, however, the same principle can become dangerous: condensates may concentrate aggregation-prone proteins, increase local supersaturation, and create conditions that favour liquid-to-solid transitions, which are increasingly implicated in neurodegeneration and cancer (Silva et al. 2023).

p53 becomes especially interesting because it is both a participant in functional nuclear condensates and a candidate scaffold for aberrant condensates. Its disordered termini, charged regions, folded but metastable DNA-binding domain, oligomerisation capacity, and affinity for DNA and RNA make it highly sensitive to the physicochemical variables that regulate phase behaviour. Protein concentration, pH, salt, redox state, temperature, pressure, nucleic acids, glycosaminoglycans, molecular chaperones, and post-translational modifications can all shift the balance among soluble p53, liquid condensates, gel-like states, and solid aggregates (Fig. 4C) (Welles et al. 2024).

Thus, before the mechanistic studies of p53 phase separation were fully developed, the field had already assembled several pieces of the puzzle: p53 is a metastable multidomain tumour suppressor; its cancer mutations frequently destabilise the DNA-binding domain; mutant p53 accumulates and forms amyloid-like aggregates in cancer; and biomolecular condensates can serve as functional compartments or pathological precursors to solid-like assemblies. The recent conceptual advance was to connect these pieces into a single model in which p53 aggregation is not an isolated endpoint, but a phase-transition process embedded in nuclear condensate biology. This perspective reframes mutant p53 as a condensate-sensitive oncogenic protein and places p53 at the crossroads between structural biology, cancer cell biology, and the physics of biomolecular organisation.

#### 4b Phase separation as the missing intermediate between p53 misfolding and oncogenic aggregation

P53 aggregation should not be viewed only as the final appearance of insoluble amyloid-like material, but as the outcome of a regulated, and potentially targetable, phase-separation pathway (Silva et al. 2023). *Petronilho *et al*.* asked a mechanistic question: does p53 aggregation arise directly from soluble monomers or oligomers, or does it pass through biomolecular condensates before acquiring gel-like and solid-like features (Petronilho et al. 2021)? The answer was that the p53C undergoes phase separation before aggregation, and that oncogenic mutations accelerate the conversion of these condensates into less dynamic amyloid-like states (Fig. 4A). This finding is central to the broader field of biomolecular condensates because it positions p53 not merely as a client of nuclear membraneless organelles, but as a protein capable of becoming an aberrant scaffold when destabilised, overproduced, or trapped in a nucleic acid-rich environment.

Mutations within p53C increased the amyloid aggregation propensity relative to the wild-type protein, as measured by thioflavin T fluorescence, reinforcing the idea that local perturbations in the DNA-binding domain can propagate into mesoscopic assembly behaviour. The key advance, however, was not only the demonstration that mutant p53C aggregates faster, but the definition of an intermediate condensate state. Under crowding conditions created with PEG, p53C demixed into droplets in a concentration- and temperature-dependent manner. At 4 °C, wild-type p53C formed liquid-like droplets and spheroids; at 15 °C, droplets and spheroids coexisted; and at 37 °C, the reaction rapidly shifted toward aggregate-like structures (Petronilho et al. 2021). This temperature-sensitive behaviour is particularly relevant because it indicates that p53 condensates are not static objects. They occupy a continuum in which the same molecular scaffold can remain dynamic, become gel-like, or harden into solid amyloid-like material depending on the physicochemical environment.

This work is also important because it disentangles the contributions of the folded DNA-binding domain and intrinsically disordered regions. Many phase-separating proteins depend primarily on low-complexity disordered regions, but p53 is more complex. Its N-terminal transactivation domain (TAD) and C-terminal regulatory region are intrinsically disordered and rich in post-translational modification sites, whereas the DNA-binding domain is folded but metastable (Fig. 4B). By comparing p53C with a p53-TAD-C construct, the authors showed that the transactivation domain (TAD) modulates both phase separation and aggregation (Petronilho et al. 2021). The TAD had previously been shown to reduce amyloid aggregation of p53C, and here it altered droplet size and condensate behaviour, suggesting that the disordered regions do not simply “cause” phase separation. Rather, they reshape the energy landscape that governs p53's transition from soluble states to condensates and then to solid-like assemblies. In this sense, p53 functions as a multidomain regulatory hub whose folded and disordered regions cooperate to determine whether condensation remains reversible or becomes pathological.

The cellular experiments provided a physiological context for the in vitro observations. Using FRAP experiments, the authors showed that p53 condensates in the nucleus recovered fluorescence more completely, consistent with liquid-like behaviour. In contrast, mutant p53, especially in nuclear bodies, showed slow and incomplete recovery, indicating gel-like or solid-like states. Thus, the nucleolus emerges as a permissive compartment in which mutant p53 can be concentrated, retained, and converted into aberrant condensates. This has direct implications for gain-of-function biology: mutant p53 may acquire oncogenic properties not only through altered DNA binding or aberrant protein interactions, but also by changing its material state within the nucleus (Petronilho et al. 2021).

Additionally, p53 phase behaviour is finely regulated by polyanions and conformational stability. RNA promoted solid-like transitions, whereas heparin stabilised more dynamic gel-like condensates and partially reversed aggregate morphology. Sub-denaturing guanidinium hydrochloride induced p53C droplets even in the absence of PEG, linking molten-globule-like destabilisation to condensation. Together, these findings position phase separation as an intermediate between p53 structural instability and amyloid aggregation. Mutant p53 may therefore enter condensate states more readily and become trapped in assemblies that mature into oncogenic amyloid-like structures, offering multiple therapeutic intervention points.

#### Mutant p53 as a prion-like catalyst of p63 and p73 condensate hardening

Further studies extended the p53 condensate model from homotypic aggregation to family-level pathological crosstalk. Could oncogenic p53 convert the condensates of its paralogs, p63 and p73, into amyloid-like assemblies? This question matters because p63 and p73 are not peripheral relatives of p53. They share structural homology within the DNA-binding domain, participate in tumour suppression and development, and can be functionally antagonised by mutant p53 in cancer. The study shows that p63C and p73C can undergo phase separation but, unlike p53C, do not readily form amyloids at physiological temperatures. Mutant p53C changes this behaviour. It recruits p63C and p73C into membraneless compartments and induces amyloid-like conversion, providing a physical mechanism for how mutant p53 may suppress the functions of p53-family proteins while gaining oncogenic activity (Fig. 4D). p63C and p73C form droplets that remain largely non-amyloidogenic under the tested physiological temperatures, whereas wild-type p53C and especially mutant p53C progress toward amyloid-like aggregation (Petronilho et al. 2024).

The M237I mutant, located near the zinc-binding region of p53C, is particularly informative because it destabilises the DNA-binding domain and greatly increases its propensity to aggregate. The authors also considered classical hotspot mutations, including R248Q, R249S, and R273H, and showed that p53 variants differ in their ability to seed the conversion of p63C and p73C droplets. R249S and M237I appeared more competent than R248Q or R273H in driving droplet-to-aggregate conversion, consistent with the idea that not all gain-of-function mutations act through the same material-state mechanism (Petronilho et al. 2024).

Combined complementary biophysical and cellular readouts defined the material-state conversion within the p53 family. DIC microscopy revealed droplet formation and morphological remodeling, whereas Congo red, thioflavin T, light scattering, and turbidity distinguished amyloid-like aggregation from reversible condensation. Circular dichroism further showed secondary-structure rearrangement, with reduced α-helical content and increased β-sheet contribution during coaggregation. Dual-label fluorescence demonstrated that p53C co-phase separates with p63C or p73C at low temperature, but at 37 °C, particularly in the presence of M237I p53C, these mixed condensates shift toward aggregate-rich states. Thus, p63C and p73C are competent to condense, but require p53-derived seeds to undergo pathological amyloid-like maturation. In cells, full-length M237I p53 colocalised with p73 and promoted p63C/p73C droplet-like assemblies in nucleolar compartments. Slow, incomplete FRAP recovery supported gel- or solid-like hardening, implicating the nucleolus as a permissive site for heterotypic p53-family phase transition (Petronilho et al. 2024).

One important finding is that heparin inhibits p53C-induced prion-like aggregation of p63C and p73C. This builds directly on the observation that heparin modulates p53 phase transition by preventing progression toward more solid-like states. Further, heparin reduced seeded aggregation and prevented the conversion of p63C droplets into amyloid-like material. Its relevance lies not in heparin itself being immediately a cancer drug in this context, but in the fact that polyanion-sensitive interfaces can be pharmacologically manipulated. The finding supports a therapeutic logic in which the target is not only a folded active site or a mutant cavity, but a pathological transition route: soluble protein > aggregation-prone monomer > condensate > gel-like state > amyloid-like aggregate (Petronilho et al. 2024).

These findings place mutant p53 as an active remodeler of the p53-family phase landscape. By recruiting p63 and p73 into heterotypic condensates and promoting their conversion into amyloid-like assemblies, mutant p53 may suppress related tumour-suppressive networks through material-state remodelling rather than through binding alone.

Similar to p53, DDX3X illustrates how disease-associated mutations can alter not only protein structure and activity, but also condensate dynamics and material state. However, the DDX3X studies additionally connect these effects to RNA helicase activity, neurodevelopment, and quantitative time-resolved measurements of condensate ageing.

### Biomolecular condensates in neurodevelopment and neurodevelopmental disorders and open research infrastructures: contributions from the Brazilian Centre for Research in Energy and Materials, CNPEM

#### DDX3X condensation and neurodevelopmental disorder (Ivan Rosa e Silva and Juliana Ferreira de Oliveira)

*Homo sapiens* DEAD-box helicase 3 X-linked (DDX3X) is an ATP-dependent RNA helicase that regulates multiple steps of mRNA metabolism and stress response. It localises to cytoplasmic RNA-rich condensates such as stress granules and neuronal transport granules, where its RNA unwinding activity modulates phase separation and the material properties of these assemblies (Silva et al. 2024). Functional and structural studies of DDX3X were initiated at the Brazilian Biosciences National Laboratory (LNBio, CNPEM) following a systematic whole-exome sequencing analysis of Brazilian patients with idiopathic syndromic intellectual disability (ID) at the Institute of Biosciences of the University of São Paulo (Carneiro et al. 2018). This study identified a heterozygous de novo pathogenic variant, p.L556S, in the *DDX3X* gene in one patient (Carneiro et al. 2018).

An integrated set of classical biophysical techniques, including light scattering, circular dichroism and fluorescence spectroscopies, NMR, and X-ray crystallography, was initially employed to investigate the impact of the L556S mutation on DDX3X structure and function, in addition to other neurodevelopmental disorder (NDD)-related mutations, including the most recurrent R376C mutation and the severe R326H mutation. It was demonstrated that both L556S and R376C mutations destabilise the DDX3X helicase domain, exposing hydrophobic surfaces and thereby promoting protein misfolding and self-assembly into amyloid-like fibrils (de Castro Fonseca et al. 2021). On the other hand, the R326H mutation resulted in minor changes to the overall protein structure while altering the chemical environment of the RNA-binding site (Rosa e Silva et al.). Importantly, each of the three NDD-associated mutations independently impaired DDX3X enzymatic activity (de Castro Fonseca et al. 2021; Rosa e Silva et al. 2026).

In neuroblastoma cells, wild-type DDX3X accumulated in stress granules while retaining the ability to exchange components with the surrounding cytoplasm, as demonstrated by FRAP experiments (de Castro Fonseca et al. 2021; Rosa e Silva et al. 2026). Conversely, DDX3X mutants exhibited lower molecular mobility within condensates in cellulo than wild-type DDX3X (de Castro Fonseca et al. 2021; Rosa e Silva et al. 2026). L556S DDX3X–positive stress granules were visible in the cytoplasm by confocal fluorescence microscopy even under normal culture conditions, appearing as dense, 1,6-hexanediol–resistant condensates capable of trapping the wild-type protein (de Castro Fonseca et al. 2021).

This scenario prompted an extensive investigation into the molecular mechanisms underlying DDX3X condensation in vitro. DDX3X phase separation is mainly driven by cation-π and π-π interactions in the RG/RGG-rich N-terminal and the G/S-rich C-terminal IDRs (Silva et al. 2024). Quantitative fluorescence microscopy experiments (Rosa e Silva et al. 2026; Rosa e Silva et al. 2025) demonstrated that wild-type, full-length DDX3X forms homotypic spherical droplets in vitro under low salt concentration. These wild-type condensates are enzymatically active in the presence of RNA (Rosa e Silva et al.). X-ray photon correlation spectroscopy (XPCS) analyses at the CATERETÊ beamline of the Brazilian 4th-generation synchrotron facility, SIRIUS, further showed that DDX3X condensates exhibit sub-second dynamics, tending to age into gel-like droplets after around 30 min (Rosa e Silva et al. 2026). In addition, homotypic condensates of purified R376C DDX3X displayed a time-dependent evolution toward elongated, aggregate-like assemblies, as observed by fluorescence microscopy (Rosa e Silva et al. 2025).

Interestingly, DDX3X homotypic condensation in vitro was shown to be finely regulated by its substrates, which mainly interact with the structured helicase domain (Rosa e Silva et al. 2026). The presence of sub-millimolar ATP or sub-stoichiometric dsRNA did not alter DDX3X condensation. The addition of low concentrations of ATP-Mg^2^⁺ to pre-formed DDX3X–dsRNA condensates led to a progressive reduction in droplet size and altered morphology, reflecting helicase-driven remodelling, an effect not observed with a non-hydrolyzable ATP analogue. In contrast, high ATP concentrations (in the low millimolar range) impaired homotypic DDX3X condensation in vitro, while stoichiometric levels of RNA similarly inhibited condensate formation (Rosa e Silva et al. 2026).

Furthermore, studies using NMR, X-ray crystallography, and molecular dynamics simulations have demonstrated that the Cl⁻ ion binds to the DDX3X helicase domain in the RNA-binding region and functionally affects the enzyme. Cl- binding competes with RNA binding, inhibiting both the ATPase and RNA helicase activities of DDX3X at physiologically relevant concentrations, as well as its ability to phase separate (Rosa e Silva et al. 2026). Cl⁻ efflux increased the size of DDX3X-containing stress granules in a neuroblastoma cell line (Rosa e Silva et al. 2026). These effects of Cl⁻ binding to DDX3X were attenuated by the NDD-linked R326H mutation (Rosa e Silva et al. 2026), which is located near the Cl⁻ binding site in the structured helicase domain. Also, R326H DDX3X condensates showed a lower relaxation time in XPCS experiments (Rosa e Silva et al. 2026).

These results suggest that Cl⁻ modulates interactions between DDX3X IDRs and the helicase domain, thereby inhibiting condensate formation. Together, the initial biophysical characterisation of DDX3X provides insights into the molecular mechanisms governing the condensation of this important human protein and how key mutations in the helicase domain modulate this process. Future studies will explore the relationship between these alterations and neurodevelopmental disorders.

Table 1 compiles the condensate-forming protein systems reviewed here (BEX3, PrP, GRASP, Grh1, engineered condensates, p53 family, and DDX3X) span diverse biological contexts, from neuronal signalling and prion diseases to Golgi architecture, cancer, and neurodevelopmental disorders. Despite this diversity, they converge on a shared set of biochemical and biophysical features: (*i*) condensation is driven by multivalent interactions involving IDRs, structured domains, and nucleic acids-biding regions; (*ii*) material states exist along a continuum spectra from dynamic liquids to gel-like and solid-like assemblies; (*iii*) physicochemical environmental modulators (pH, ions, crowding, ligands) and post-translational modifications finely tune phase behaviour; and (*iv*) disease-associated mutations can shift the phase boundary towards solidified pathologically-linked states.

**Table 1:** Condensate-forming protein systems studied by the Brazilian phase separation community

Protein	Main Driver(s)	Key Modulators	Summary of Observed Material State	References
Brain Expressed X-linked 3 (BEX3)	IDR + coiled-coil domain + nucleic acid binding regions	tRNA fragments (modulate LLPS, ↓ amyloid); Temperature (reversible hydrogel)	Hydrogel (reversible); Liquid droplets; Viscous gels; Amyloid-like aggregates; Soluble oligomers	(do Amaral et al. 2020; Cabral et al. 2015)
PrP	N-term IDR (octapeptide repeats) + nucleic acid binding regions	Nucleic acids (DNA aptamers, RNA); Divalent metal ions; Oxidation; Ionic strength; pH; Crowding; Temperature	Liquid-, gel-like and solid-like droplets; Liquid-to-solid transition; Amyloid-like aggregates	(do Amaral et al. 2023; Matos et al. 2020; do Amaral et al. 2023; do Amaral et al. 2025; Tahira et al. 2025)
GRASP (GRASP55/65; Grh1)	IDRs; Serine-proline-rich region	pH; Membrane environment; Lipid composition; Myristoylation; Interfacial hydration	Liquid-, gel-like and solid-like droplets; amyloid-like aggregates	(Mendes et al. 2024b; Mendes et al. 2024a; Oliveira et al. 2026; Fontana et al. 2018)
Chimeric Engineered Condensates	LCD from Dhh1; Catalytic domain (Pp 1,2-CCD)	Crowding; Time-dependent maturation; Environmental conditions	Liquid droplets; Solid/irreversible assemblies	(Evangelista et al. 2024)
p53 family	DBD/p53C (folded but metastable); TAD and C-term IDRs	Mutations (M237I, R248Q, R249S, R273H); Temperature; Crowding; Polyanions (RNA, Heparin); GdnHCl	WT: Liquid droplets; Mutant: Amyloid fibrils, gel/solid; p63/p73: Liquid droplets; Amyloid upon mutant seeding	(Silva et al. 2023; Galarreta et al. 2025; Ano Bom et al. 2012; Pedrote et al. 2020; Petronilho et al. 2021; Petronilho et al. 2024)
DDX3X	RG/RGG-rich N-term IDR + G/S-rich C-term IDR; helicase domain; RNA binding	NDD mutations (L556S, R376C, R326H); ATP; RNA; Cl⁻ ions; Time (aging); Salt	WT: Liquid droplets; L556S/R376C: Amyloid-like fibrils, solid aggregates; R326H: Slower dynamics	(Silva et al. 2024; de Castro Fonseca et al. 2021; Rosa e Silva et al. 2026; Rosa e Silva et al. 2025)

#### Community-oriented infrastructures to advance biomolecular condensation research in Brazil (Aline Ribeiro Passos and Ivan Rosa e Silva)

Quantitative biophysical methods designed to study biomolecular phase separation in vitro provide a powerful framework for dissecting condensate behaviour under controlled conditions (Rosa e Silva et al. 2025). Such methods enable systematic assessment of condensate formation, morphology, and internal dynamics, offering quantitative insight into the physical principles governing biomolecular condensate assembly.

In particular, image-based assays enable the construction of time-resolved phase diagrams and the quantification of molecular partitioning in defined biomolecular systems, while allowing systematic evaluation of the sensitivity of phase behaviour to environmental and biochemical perturbations (Rosa e Silva et al. 2025). Comparative analyses across mutants, post-translationally modified proteoforms and binding partners further enable the identification of underlying interaction motifs and support the development of predictive, quantitative models. Together, these approaches establish a direct link among protein sequence, emerging biophysical properties, physiological functions, and their contributions to disease.

Researchers at the LNBio-CNPEM have recently developed an advanced image-based platform for the quantitative characterisation of biomolecular phase separation (Pedrote et al. 2020), which is available to the biophysical community through the Bioassays Facility. This approach integrates plate-based high-content imaging of biomolecular condensates using the Operetta CLS imaging system (PerkinElmer) with automated analysis to enable robust mapping of time-resolved phase diagrams, quantification of molecular partitioning, and determination of phase boundaries under controlled conditions (Rosa e Silva et al. 2025). This approach has recently been applied to the study of human DDX3X condensation in vitro (Rosa e Silva et al. 2026; Rosa e Silva et al. 2025), as further described in Section "DDX3X condensation and neurodevelopmental disorder (Ivan Rosa e Silva and Juliana Ferreira de Oliveira)".

**Fig. 5:**
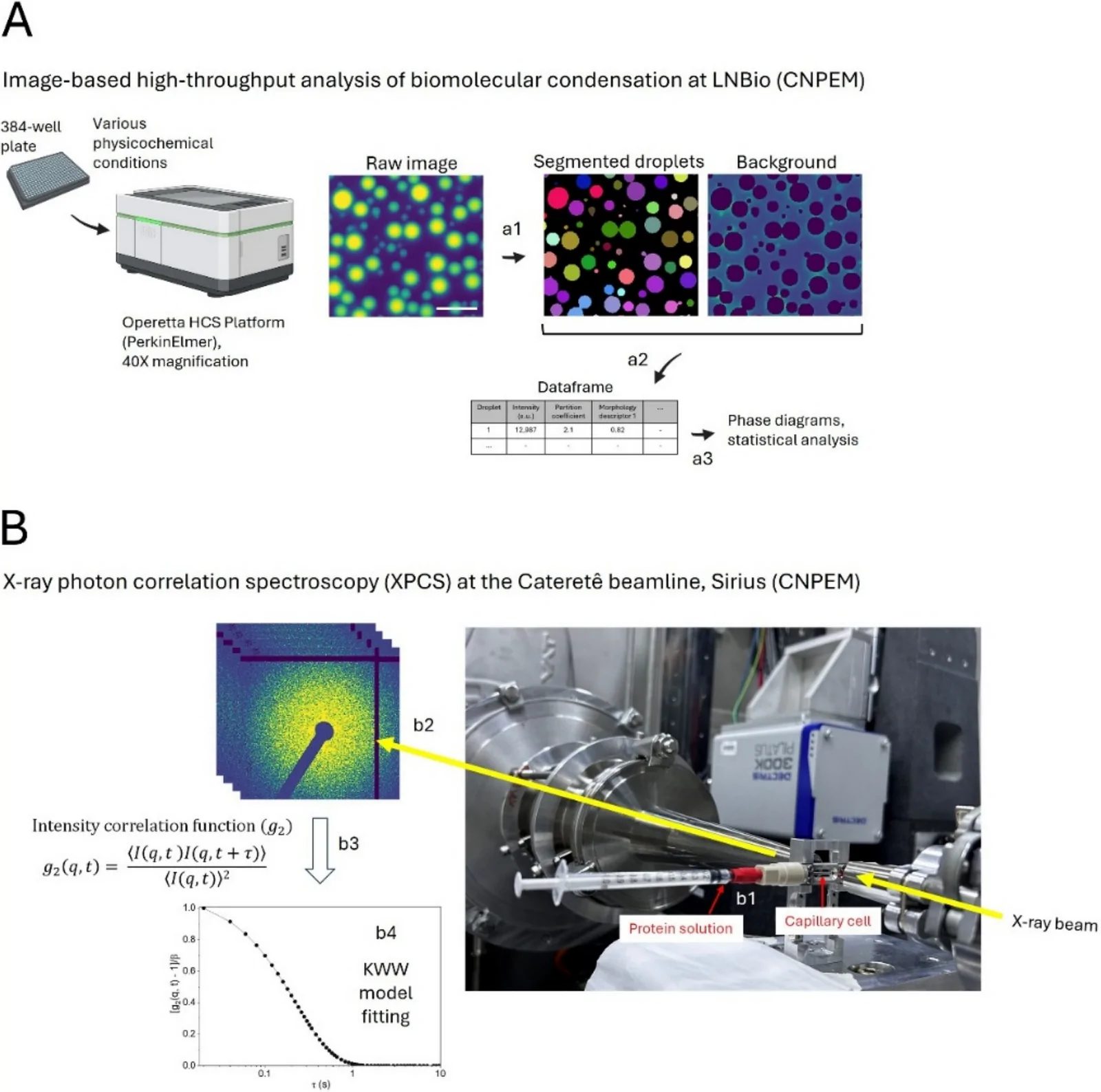
Biophysical methods for the quantitative in vitro characterisation of biomolecular condensates. **A** Image-based quantification of DDX3X droplet morphology and biomolecular partition at LNBio/CNPEM. (a1) Raw fluorescence microscopy images are segmented after normalisation, denoising, and enhancement. (a2) Fluorescence intensity of individual droplets is quantified by applying a refined binary mask to the original images. Background regions are defined as the inverse of the binary mask. (a3) Morphological features are then extracted, and the resulting data are organised into a structured dataset, incorporating experimental metadata, for phase diagram construction and downstream statistical analysis. **B** X-ray photon correlation spectroscopy (XPCS) measurements of biomolecular condensates performed at the experimental hutch of the CATERETÊ beamline (Sirius, LNLS/CNPEM). (b1) The protein solution is loaded into a capillary using a syringe, and (b2) speckle patterns are recorded by a detector located inside a vacuum chamber. (b3) The intensity autocorrelation function (${g}_{2}$) is calculated as a function of the time delay. (b4) The protein dynamics is characterised by the exponential decay of the ${g}_{2}$ function

Image analysis (Fig. 5A) is enabled through a robust and accessible Python-based computational pipeline (Rosa e Silva et al. 2025), delivered within an interactive Jupyter notebook environment, that facilitates droplet segmentation and quantitative characterisation of morphological heterogeneity in biomolecular condensate assays. In addition to conventional morphology descriptors such as roundness and circularity, the image analysis pipeline incorporates advanced morphological descriptors, including the Euler characteristic and fractal dimension, which are sensitive to subtle spatial features. Beyond its analytical capabilities, the pipeline features an interactive module for phase diagram construction that supports real-time visualisation and concise summarisation of quantitative results. The workflow also integrates statistical analyses to systematically evaluate the distribution of morphological data. Furthermore, the inclusion of multivariate techniques, such as interactive principal component analysis (PCA), complemented by correlation matrices and scree plots, provides a comprehensive approach for identifying complex patterns and relationships in high-dimensional datasets (Rosa e Silva et al. 2025).

While compositional approaches, such as the imaging technique described, reveal which biomolecules are enriched in condensates, complementary low-throughput biophysical methods provide a toolkit for quantifying material properties and mesoscale organisation of the biomolecules. For instance, XPCS experiments (Fig. 5B) allow probing collective dynamics, viscoelasticity, and nanostructure of biomolecular condensates. Biomolecular condensates are complex fluids under non-equilibrium conditions that can undergo gelation or solid-like transitions, leading to dynamic arrest. Accessing the dynamics of these condensates is essential for understanding molecular motion within the condensates, their fluidity, and phase transitions. XPCS is well-suited for such biophysical studies, as it probes a broad range of time and length scales (Perakis and Gutt 2020).

XPCS relies on coherent X-rays, and the high coherent photon flux provided by fourth-generation synchrotron sources has significantly expanded its range of applications. At Sirius, XPCS is currently available at the CATERETÊ beamline and is being commissioned at the SAPUCAIA beamline. Given the weak scattering and radiation sensitivity of proteins, dedicated experimental protocols have been developed to enable reliable XPCS measurements of biomolecular condensates at the CATERETÊ beamline (do Amaral et al. 2023; do Amaral et al. 2025; Rosa e Silva et al. 2026). Briefly, XPCS experiments are performed using capillary-based sample environments with temperature control. Prior to the XPCS measurements, the radiation-damage threshold dose is determined from the scattering profile of the protein sample in solution as X-ray exposure increases. Data analysis is performed using a Python-based graphical user interface within an interactive Jupyter notebook environment (https://doi.org/10.5281/zenodo.13629240), supporting the optimisation of correlation analysis parameters. The resulting correlation functions are fitted using the Kohlrausch–Williams–Watts (KWW) function, providing the relaxation rate and the KWW exponent associated with the nature of the dynamics:

1 $${g}_{2}(q,t)= {1+ \beta e}^{-2{(t/\tau (q))}^{\gamma (q)}}$$

where *β* is the speckle contrast, *τ* the relaxation time, and *γ* is the KWW exponent, reflecting the nature of the underlying dynamical processes, where *γ* = 1 is related to simple diffusive Brownian motion, *γ* < 1 to subdiffusive dynamics, and *γ* > 1 to superdiffusive dynamics. The XPCS analysis pipeline at Sirius was developed to be user-friendly, making it accessible to the biophysics community. The molecular dynamics probed by XPCS provide quantitative information on the physical state of protein condensates and their transitions. In addition, structural information can also be obtained simultaneously by SAXS from the average scattering intensity of the XPCS speckle patterns.

The effects of chemical perturbations on biomolecular condensates, such as the presence of copper ions and hydrogen peroxide, can be directly probed by XPCS, revealing significant changes in their dynamic properties, as observed in recent in vitro studies of prion protein condensates, as further discussed in Sect. "2a PrP and nucleic acids" (do Amaral et al. 2023). Also, the time-resolved maturation of condensates can be studied in detail, as shown for the homotypic condensation of DDX3X discussed in Section "DDX3X condensation and neurodevelopmental disorder (Ivan Rosa e Silva and Juliana Ferreira de Oliveira)" (Michaels et al. 2023), where a gradual shift toward slightly superdiffusive behaviour was observed, accompanied by increasing relaxation times, consistent with droplet growth. Together, these recent examples highlight XPCS's ability to capture protein condensate dynamics and fluidity.

### Contributions from other Brazilian groups

In parallel to the research described by the contributing authors’ groups, several other Brazilian groups have advanced the field by connecting condensate formation to concrete mechanistic questions in signalling, stress adaptation, and host–pathogen biology, while also proposing physics-driven conceptual frameworks.

In cell signalling, the group led by Fernando A. Melo (São Paulo State University) reported that Grb2 can behave as a switchable condensate-forming scaffold whose material state depends on its oligomeric state (Dias et al. 2026). In their work, the auto-inhibited Grb2 homodimer forms only transient assemblies, whereas a phosphorylation-mimetic monomer (Y160E) robustly phase-separates and matures into gel-like condensates that can recruit wild-type Grb2 as a client, suggesting a controllable scaffold–client mechanism for building high-density signalling hubs (Dias et al. 2026).

At the interface between proteostasis and stress biology, the groups led by Aline M. Santos and André Thomaz (University of Campinas) proposed a nuclear quality-control role for condensates in cardiomyocytes subjected to doxorubicin-induced oxidative stress (Aparecida Moretto et al. 2025). They identified Bclaf1 as a PTK2 interactor and showed that Bclaf1 forms nuclear biomolecular condensates in which PTK2 is sequestered and protected from ubiquitin–proteasome-mediated degradation, with downstream consequences for p53 regulation and cell survival (Aparecida Moretto et al. 2025).

In viral systems, the group led by Fabio C. L. Almeida (Federal University of Rio de Janeiro) showed that the SR-rich motif in the N-terminal domain of the SARS-CoV-2 nucleocapsid protein (N-NTD-SR) is required for nucleic acid-driven condensation under macromolecular crowding. Additionally, condensation depends on the stoichiometry, structure and binding specificity of short DNA oligonucleotides, specifically, single-stranded (+) or (-) and double-stranded (ds) transcriptional regulatory sequences (TRS). The high-affinity duplex TRS triggers N-NTD-SR phase separation at a lower protein:DNA stoichiometry than the single-stranded counterparts. They also argue that N-NTD-SR melting activity cannot be explained by DNA-binding affinity alone but also by binding geometry. This is supported by their NMR data and MD simulations in which the dsTRS is positioned and oriented on the N-terminal binding surface compared with a non-specific duplex (dsNS). Notably, they report an ~ 25° difference in orientation, along with distinct contacts between the TRS triple‑thymidine (TTT) motif and a defined β‑sheet region. Consistent with these distinct interaction geometries, they observe qualitative differences in the resulting phase-separated assemblies, with dsNS conditions favouring wetting and, in some regimes, more solid-like/crystalline material. In contrast, dsTRS condensates remain more liquid-like (Caruso et al. 2022).

At a broader conceptual scale, the group led by Francisco Prosdocimi (Federal University of Rio de Janeiro) revisited the classical coacervate theory. They proposed a reinterpretation aligned with modern phase separation, arguing that early biological compartmentalisation could have emerged from phase separation of intrinsically disordered peptides and RNAs (Prosdocimi and Farias 2025). They frame LLPS-based protocompartments as a plausible route to concentrate reactants and create localised microenvironments in prebiotic settings, integrating this view into a ribonucleoprotein-world hypothesis for early evolution (Prosdocimi and Farias 2025). Condensates offer unique advantages, such as precise spatiotemporal control of cellular reactions, which likely shaped the early prebiotic trajectory of life. Importantly, initially condensates were a means to concentrate molecules, but over time, complex regulatory mechanisms have evolved to control their assembly/disassembly.

In a theoretical work, the group led by Mariano de Souza (São Paulo State University) proposed a physics-driven interpretation of intracellular compartmentalisation by introducing a cellular Griffiths-like phase (CGLP) as an analogue of Griffiths phases, in which “rare regions” coexist within a distinct background (Squillante et al. 2024). In their biological mapping, randomly distributed protein droplets act as protein-rich rare regions embedded in a dilute matrix, naturally represented in a temperature–protein-concentration phase diagram with a binodal/spinodal structure. Building on Flory–Huggins solution theory as a working model, they use thermodynamic response functions to argue that proximity to phase boundaries should be detectable via anomalies in these quantities, and they connect this thermodynamic proximity to slow relaxation modes by proposing that the characteristic diffusion/relaxation time becomes strongly enhanced near the binodal (and at specific composition regimes in their analysis). A key conceptual point is that such a slow-mode regime can, in principle, couple compartmentalisation to reduced concentration noise, motivating the CGLP as a compact framework for discussing droplet heterogeneity and “critical-like” behaviour in cells without requiring that all observations be interpreted solely through an ideal-liquid LLPS lens (Squillante et al. 2024).

The group led by Claudia Mermelstein (Federal University of Rio de Janeiro) characterised the protein composition of the perinuclear region using membrane-extracted TEM, immunofluorescence, and database-driven in silico analyses, revealing a high enrichment of intrinsically disordered proteins with predicted propensity for phase separation. Their findings suggest that the perinuclear space functions as a specialised cellular hub for the assembly of biomolecular condensates, advancing the understanding of how membrane-associated regions contribute to intracellular organisation in eukaryotic cells (do Amaral et al. 2022).

### Convergent themes emerging from Brazilian condensate research

Taken together, the studies discussed above reveal a set of recurring principles that extend beyond the individual proteins, biological systems, and research groups involved. A first convergent theme is that biomolecular condensation is strongly shaped by molecular context. Nucleic acids, metal ions, post-translational modifications, pH, ionic composition, macromolecular crowding, protein concentration, and conformational stability do not merely determine whether condensation occurs. They also regulate condensate composition, internal organisation, molecular mobility, and propensity to undergo further material-state transitions. In BEX3, tRNA fragments alter protein conformation, condensate abundance, and aggregation morphology. In PrP, RNAs, DNA aptamers, Cu^2^⁺, and Zn^2^⁺ produce distinct condensation and aggregation outcomes. Grh1 and mammalian GRASPs are regulated by pH, membrane anchoring, and N-terminal modifications, whereas DDX3X condensation is modulated by RNA, ATP, chloride ions, and disease-associated mutations. p53 phase behaviour is similarly sensitive to nucleic acids, molecular crowding, destabilisation, and oncogenic mutations. Across these systems, ligands and environmental variables act not simply as passive cofactors, but as active determinants of condensate formation, organisation, dynamics, and maturation.

A second common principle is that condensates occupy a continuum of material states rather than a binary distinction between dispersed proteins and ideal liquid droplets. Brazilian studies have documented liquid-like assemblies, viscoelastic condensates, hydrogels, arrested states, amyloid-like structures, and persistent aggregates. BEX3 condensates can evolve toward fibrillar or amorphous assemblies depending on the presence of tRNA fragments. PrP condensates can remain dynamic, inhibit or delay amyloid formation, or undergo oxidation-driven liquid-to-solid transitions. Grh1 condensation may protect the protein from aggregation-prone intermediates under physiological stress conditions, while prolonged stress and environmental modulators promote condensate maturation and fibrillation. In p53, phase separation precedes aggregation, and oncogenic mutations accelerate the conversion of dynamic condensates into gel- and solid-like states. DDX3X condensates likewise display time-dependent ageing, with disease-associated variants showing reduced mobility and increased propensity for aberrant assembly. These observations collectively reinforce that the biologically relevant question is often not simply whether condensation occurs, but which material state is generated, how long it persists, and whether it remains reversible.

A third convergent theme is the context-dependent relationship between condensation and biological function or dysfunction. Condensation can support adaptive organisation by concentrating cargoes, regulating biochemical reactions, facilitating unconventional secretion, buffering potentially harmful metal ions, or creating synthetic enzymatic microreactors. The same physicochemical process, however, can become deleterious when mutations, oxidative stress, altered ligand stoichiometry, or prolonged environmental perturbation shift condensates toward persistent states. This duality is particularly evident in PrP, whose condensates may buffer redox-active copper while becoming aggregation-prone under oxidative conditions, and in Grh1, whose condensation can be protective but may also precede amyloid formation. In p53 and DDX3X, disease-associated mutations alter condensate dynamics and promote aberrant material states linked to cancer or neurodevelopmental disorders. These findings argue against assigning an intrinsically beneficial or pathological role to condensation. Instead, biological outcome emerges from the coupling among molecular composition, cellular location, material properties, and regulatory context.

Finally, the Brazilian contributions converge methodologically in their use of complementary approaches to connect molecular interactions with mesoscale behaviour. NMR, structural biology, mutational analysis, fluorescence microscopy, FRAP, phase-diagram mapping, SAXS, XPCS, computational modelling, and automated image analysis have been combined to investigate condensate formation, composition, dynamics, and maturation. This multi-method strategy is particularly important because no single experimental hallmark is sufficient to establish phase separation or define material state. The development of open computational pipelines and the use of shared national research infrastructures have further enabled quantitative and reproducible measurements across distinct condensate systems.

## Anderson de Sá Pinheiro’s contributions to the biomolecular condensates field in Brazil and beyond

### A tribute by Yraima Cordeiro

In this section, I describe my scientific collaboration with Anderson S. Pinheiro and highlight his contributions to advancing research on protein phase separation in Brazil. The collaboration between our groups emerged from the combination of complementary expertise. While my research group has long focused on protein aggregation, prion protein biology, and protein–ligand interactions, Pinheiro’s group specialised in protein NMR in solution and in the structural characterisation of protein–nucleic acid interactions. Together, we aimed to address a structural question: determining the three-dimensional structure of a complex formed between recombinant prion protein (PrP) and a DNA aptamer previously selected by SELEX in my laboratory. The PhD student Carolina O. Matos conducted this project.

However, all attempts to obtain a soluble protein–nucleic acid complex were unsuccessful. Upon mixing the components, the solution rapidly became turbid, preventing the acquisition of well-resolved peaks in ^15^N-HSQC NMR spectra of isotopically labelled PrP. At that time, these observations were initially interpreted as a technical obstacle that prevented structural characterisation of the complex.

The turning point came in late 2018 during the Annual Meeting of the Brazilian Society for Biochemistry and Molecular Biology (SBBf). While attending a symposium on ‘Structural and Unstructural Biology’ chaired by Dr Rosangela Itri, we heard a lecture by Vladimir Uversky on the unusual biophysics of intrinsically disordered proteins (IDPs). At that moment, we realised that the turbidity we had repeatedly observed in our experiments was not merely an experimental artifact but rather consistent with phase separation induced by the interaction between prion protein and DNA aptamers.

This insight redirected our efforts toward investigating and characterizing NA–driven phase separation of PrP. The work culminated in our first publication on the subject, with major contributions from Mariana Juliani do Amaral, who played a central role in the experimental characterisation of this phenomenon.

During the same period, Pinheiro’s group also made important contributions to understanding biomolecular condensates involving RNA-binding and viral proteins. His laboratory investigated in detail the interaction between the nucleocapsid (N) protein of coronaviruses, including SARS-CoV-2, and nucleic acids (Caruso et al. 2022; Caruso et al. 2021). These studies resulted in two original publications, one of which, in collaboration with Fabio C. L. Almeida at the Brazilian National Center for Nuclear Magnetic Resonance (CNRMN), elucidated the molecular mechanisms underlying nucleic acid–mediated phase transitions of the viral N protein.

In addition to these studies, Anderson Pinheiro contributed significantly to the understanding of RNA-binding proteins involved in plant responses to environmental stress. His work on the role of phase separation in plant biology culminated in a review article published in the *Journal of Biological Chemistry* in 2025 (Pougy et al. 2025), which discussed phase separation as a key mechanism in plant development, environmental adaptation, and abiotic stress responses. This line of research continues through the work of his students, Karina Pougy and Giovanna Melo, reflecting the lasting impact of his scientific vision and mentorship (Melo et al. 2026).

Through these contributions, Anderson S. Pinheiro helped establish and expand the study of biomolecular condensates in Brazil, bridging structural biology, nucleic acid biochemistry, and protein phase behaviour. His collaborative spirit, scientific rigour, and intellectual curiosity played an important role in advancing this emerging field both nationally and internationally.

### A tribute by Mariana J. Do Amaral

Anderson de Sá Pinheiro was a terrific PhD advisor and remains a role model in mentorship, profoundly shaping my career and that of many other scientists. From avidly teaching structural biology to writing precise and meaningful recommendation letters to enthusiastically discussing phase separation to even contributing hands-on at the bench when needed, his dedication to research, training, and teaching is remarkable.

Anderson’s lab has focused on RNA-binding proteins, and he hypothesised that AtGRP2, a glycine-rich protein from *Arabidopsis thaliana* that interacts with RNA, could undergo phase separation. This protein contains an N-terminal cold shock domain with two zinc finger motifs. In 2019, we established a collaboration to investigate its phase separation properties. Bioinformatic analysis using catGRANULE (Monti et al. 2025) predicted a high propensity for condensate formation (catGRANULE score: 6.9), exceeding that of FUS (5.8). Indeed, purified full-length AtGRP2 forms condensates, likely mediated by two glycine-rich regions identified as low-complexity prion-like segments using the PLAAC (Prion-Like Amino Acid Composition) algorithm. These findings were presented at the SBBF 2019 meeting by Clara M. L. F. da Motta, a talented Master’s and later PhD student who was also crucial to catalysing further studies on the phase behaviour of RNA-binding proteins in Pinheiro’s lab.

During the pandemic, Pinheiro’s group joined forces with Marcius Almeida's lab to characterise the phase separation of the SARS-CoV-2 nucleocapsid (N) protein. I was invited to join this LLPS subgroup, part of a larger Brazilian collaborative effort led by Fabio C. L. Almeida in 2020, which aimed to determine the structural and biophysical properties of SARS-CoV-2 proteins. Our work demonstrated that an N-terminal 31-amino-acid region (residues 176–206), enriched in serine and arginine (SR-rich), is critical for RNA-driven LLPS of the SARS-CoV-2 N protein under macromolecular crowding conditions (Caruso Í et al. 2021). This work was later published in 2022, the same year Anderson established a phase-contrast microscope with epifluorescence in his lab through the SARS-CoV-2 project, a setup I helped configure, which subsequently accelerated several lines of research.

Studying phase separation has only strengthened, rather than separated, our collaboration. The only “liquid-like” aspect of this partnership has been the coalescence of ideas, culminating in a dense and dynamic exchange and the launch of the first seminar series in Brazil, Phase Transitions of Biomolecules. The series kicked off on April 18, 2022, with a Zoom presentation by Marcius Almeida, whose group was the first to demonstrate, using DIC microscopy, in vitro condensate formation by recombinant BEX3, an intrinsically disordered RNA-binding protein (do Amaral et al. 2020). Today, we have a WhatsApp group that serves as a hub for sharing research on biomolecular condensates within the Brazilian community, with 63 members (as of May 2026).

Anderson has been an inspiring figure for generations of scientists, pairing an exceptional standard of scientific rigour with a genuine humility that defines both his work and his character. He has profoundly shaped the lives of many students who have had the opportunity to learn from him through passionate mentorship, thoughtful guidance, and a remarkable enthusiasm for science. Beyond his individual impact, he has also played a key role in bringing the Brazilian biomolecular condensate community together. His ideas and research will undoubtedly resonate, inspiring the next generation of scientists.

## Strengths, weaknesses, and outlook: latest developments, opportunities, and challenges for the next decade

**Fig. 6:**
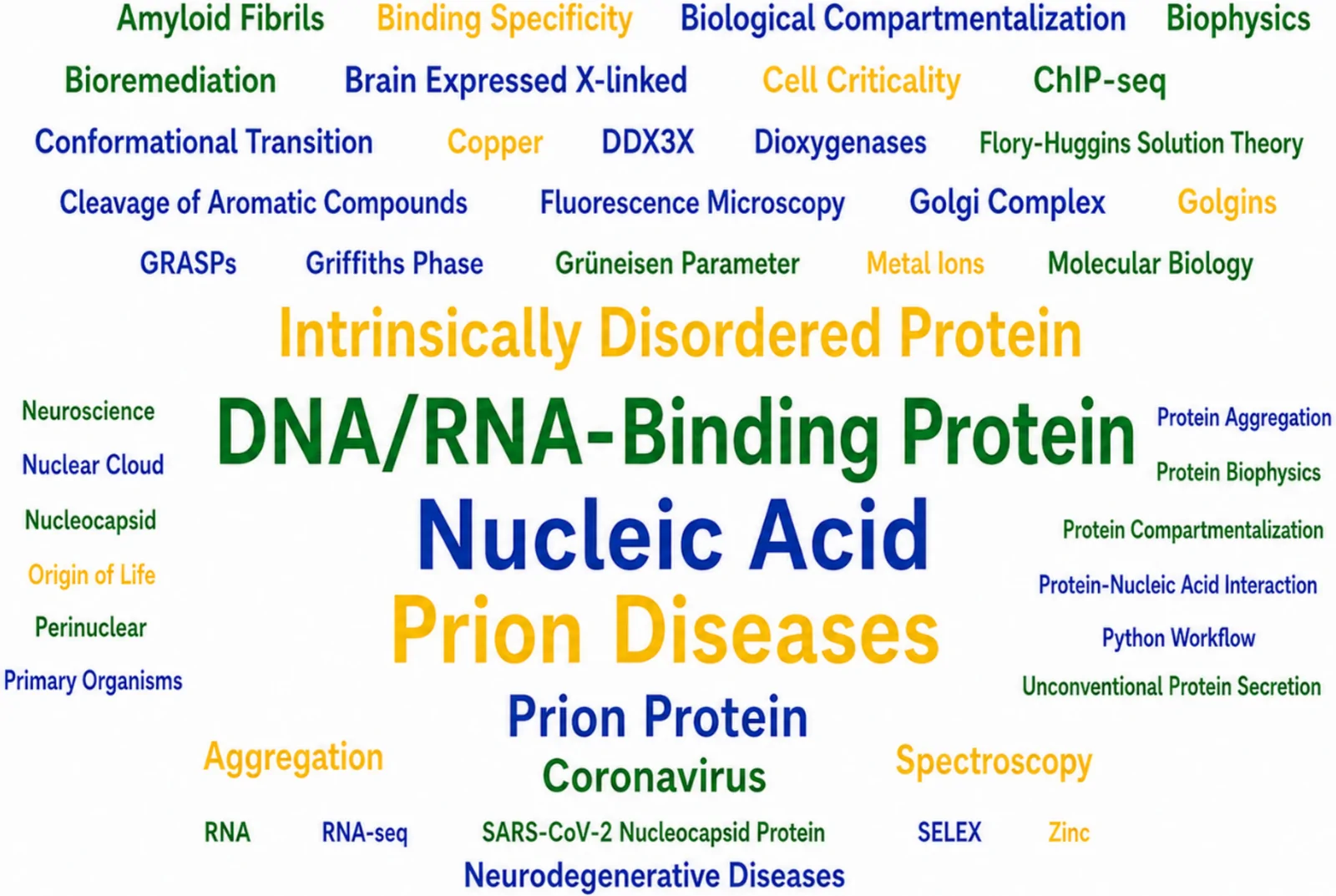
Overview of biomolecular condensate studies led by Brazilian groups, highlighting the diversity of biological systems, molecular targets, and disease-related processes investigated. Word cloud with keywords extracted from the selected publications using VOSviewer, manually curated to combine synonymous terms, and weighted according to their frequency of occurrence. Terms that directly describe phase separation phenomena (e.g., LLPS, liquid–liquid phase separation, and biomolecular condensates) were intentionally excluded to highlight the broader scientific themes explored by the Brazilian community. The curated keyword list and frequency data were provided as input to ChatGPT, which was used to generate the final word cloud visualisation. Word size is proportional to keyword frequency

The next decade of biomolecular condensate research will likely be defined by a shift from a descriptive phase to the quantitative assessment of mechanisms. The field has already moved beyond the simple identification of round, fusion-prone “droplets” and now faces more challenging questions: how do specific molecular interactions generate distinct material states, and how do these states give rise to biological function or dysfunction? This transition is particularly important to our small Brazilian community, whose contributions already span protein misfolding, neurodegenerative and neurodevelopmental disorders, cancer, viral proteins, Golgi biology, unconventional secretion, membrane-associated condensation, enzymatic microreactors, and quantitative biophysical platforms, including 22 original papers (PubMed search on May 20, 2026) (Fig. 6).

The first major priority is to move from qualitative classification of condensate states toward quantitative and time-resolved measurements of material-state transitions. The studies discussed above establish that reversibility, ageing, and dynamic arrest are central variables across diverse biological systems. Future work should therefore combine measurements of molecular mobility, viscoelasticity, composition, and structural maturation with functional readouts, allowing phase behaviour to be connected directly to biological outcomes.

A second priority is to bring condensate studies closer to physiological complexity. Many in vitro studies have been essential for establishing causality, but cellular condensates are shaped by active processes, competing interactions, spatial confinement, membranes, post-translational modifications, and local biochemical gradients. Membrane-associated condensation is especially promising. Recent work on Golgi matrix proteins indicates that condensates and membranes can be physically coupled, with condensates wetting, deforming, or reorganising lipid bilayers, while membranes impose two-dimensional constraints and serve as nucleation surfaces (Mendes et al. 2024b; Rebane et al. 2020; Zhang and Seemann 2024). This is particularly relevant for organelles such as the Golgi apparatus, where architecture, secretion, and membrane identity are inseparable. Over the next decade, reconstituted systems that combine purified proteins at physiologically relevant concentrations, defined lipids, post-translationally modified proteoforms, and controlled geometry will be essential for bridging the gap between in vitro test-tube assays and cellular organisation.

A third frontier is the integration of condensates with disease mechanisms. The Brazilian literature already provides strong examples: PrP condensates may buffer metal ions but also undergo oxidation-driven solidification; DDX3X mutations alter enzyme activity and condensate dynamics in neurodevelopmental disease; mutant p53 can use condensate states as intermediates toward oncogenic amyloid-like aggregation; and viral proteins exploit nucleic-acid-dependent condensation to organise replication-related processes. These studies suggest that condensates can be both protective and deleterious, depending on context. It is also clear that the condensates, once thought of as crucibles for pathological aggregation, constitute a non-canonical misfolding pathway that might also suppress fibril formation. Interestingly, condensates can prevent the template-assisted protein misfolding or sequester misfolded oligomers of aggregation-prone proteins (Lipiński et al. 2022; Mahendran et al. 2026). Recent studies using different condensate “driver” proteins have shown a direct link between the nature and stoichiometry of “client” ligands and small molecules (Lipiński et al. 2022; Mahendran et al. 2026). These factors are crucial for tuning condensate metastability and for promoting or suppressing aggregation. For instance, PrP pre-formed Cu^2+^ or Zn^2+^-driven condensates prevent or delay, respectively, the polymerisation of recombinant PrP into ThT-positive amyloid-like species (do Amaral et al. 2023; Juliani do Amaral et al. 2025). We are also just beginning to understand that condensates can be highly heterogeneous, exhibiting interiors with properties opposite to those of their surfaces (Shen et al. 2023). Another major challenge will be to distinguish adaptive condensation from pathological phase transitions in cells and tissues. This will require perturbation experiments, endogenous expression systems, quantitative imaging, proteomics, transcriptomics, and functional assays to test whether altering condensate material properties affects disease-relevant phenotypes.

A major limitation in most studies in our community is the difficulty of directly measuring condensate properties in relevant cell models. Additionally, we now appreciate that most condensates are non-stoichiometric assemblies formed by a myriad of “driver” scaffold proteins co-mixed with “clients” like nucleic acids and other biomolecules, as shown by the “condensome” of phase-separated compartments. Further directions in the field could involve developing mass spectrometry methods tailored to the dynamic, versatile nature of endogenous cellular condensates.

A fourth opportunity lies in engineering. Condensates are no longer only objects of cell biology; they are becoming design principles for synthetic microreactors, artificial organelles, and programmable biomaterials. The development of condensate-based enzymatic platforms for the degradation of environmental pollutants illustrates how phase separation can be used to concentrate catalysts, tune reaction environments, and produce active, dense phases with technological value. Future applications may include metabolic channelling, biosensing, enzyme stabilisation, drug delivery, and synthetic control of cellular pathways. However, this direction will require rigorous control over reversibility, ageing, specificity, permeability, and long-term stability.

For Brazil, a major opportunity lies in community-level integration. This is not only scientifically desirable, given the interdisciplinary nature of condensate research, but also strategically important in a research environment constrained by limited funding, insufficient postgraduate fellowships, and delays in access to imported reagents and equipment. Nonetheless, our Brazilian community has complementary strengths in molecular biophysics, structural biology, spectroscopy, NMR, SAXS, XPCS, fluorescence microscopy, cell biology, computation, and synchrotron-based methods, perfectly fitting the intrinsically interdisciplinary nature of the field. The availability of shared research resources such as Sirius beamlines (https://lnls.cnpem.br/sirius-en/, accessed in 2026) and LNBio/CNPEM high-content imaging platforms (https://lnbio.cnpem.br/en/instalacoes-abertas/bioensaios/, accessed in 2026) creates a distinctive national advantage. XPCS, in particular, can provide access to condensate dynamics over time and length scales that are difficult to capture by microscopy alone, while automated imaging and open computational pipelines can support reproducible phase diagrams and comparative datasets, including high-throughput screening of biomolecular condensation modulators. Access to these examples of high‑end techniques is especially important, as they would otherwise be unavailable within the inadequate infrastructure on which most public universities rely. Key initiatives are urgently needed to further strengthen the condensates community in Brazil and across Latin America. A primary driving force behind the advancement of our community is the research conducted by PhD and Master’s students. Ensuring sufficient fellowships is therefore critical, alongside the necessary supplies and equipment that could be supported through national‑ and regional‑level funding programs specifically dedicated to biomolecular condensates.

The main challenges facing the global biomolecular condensates community are equally evident (Alberti et al. 2025). The field needs shared standards for experimental evidence, careful language to avoid overinterpreting liquid-like behaviour, better quantification of material properties, and stronger connections between in vitro phase diagrams and cellular function. It also needs training programs that can cross disciplinary boundaries, from polymer physics to molecular and cell biology. If our Brazilian community can combine rigorous methodology, collaborative infrastructure, and mechanistic ambition, it is well-positioned not only to follow international developments but also to shape the understanding of condensates as dynamic, regulated, and functional states of biological matter.

## Data Availability

No datasets were generated or analysed during the current study.
